# Mesocorticolimbic monoamine correlates of methamphetamine sensitization and motivation

**DOI:** 10.3389/fnsys.2014.00070

**Published:** 2014-05-07

**Authors:** Kevin D. Lominac, Courtney L. McKenna, Lisa M. Schwartz, Paige N. Ruiz, Melissa G. Wroten, Bailey W. Miller, John J. Holloway, Katherine O. Travis, Ganesh Rajasekar, Dan Maliniak, Andrew B. Thompson, Lawrence E. Urman, Tamara J. Phillips, Karen K. Szumlinski

**Affiliations:** ^1^Department of Psychological and Brain Sciences, Neuroscience Research Institute, University of California at Santa BarbaraSanta Barbara, CA, USA; ^2^Behavioral Neuroscience, Methamphetamine Abuse Research Center, Veterans Affairs Medical Center, Oregon Health and Science UniversityPortland, OR, USA

**Keywords:** methamphetamine, sensitization, nucleus accumbens, dopamine, prefrontal cortex, serotonin, addiction vulnerability

## Abstract

Methamphetamine (MA) is a highly addictive psychomotor stimulant, with life-time prevalence rates of abuse ranging from 5–10% world-wide. Yet, a paucity of research exists regarding MA addiction vulnerability/resiliency and neurobiological mediators of the transition to addiction that might occur upon repeated low-dose MA exposure, more characteristic of early drug use. As stimulant-elicited neuroplasticity within dopamine neurons innervating the nucleus accumbens (NAC) and prefrontal cortex (PFC) is theorized as central for addiction-related behavioral anomalies, we used a multi-disciplinary research approach in mice to examine the interactions between sub-toxic MA dosing, motivation for MA and mesocorticolimbic monoamines. Biochemical studies of C57BL/6J (B6) mice revealed short- (1 day), as well as longer-term (21 days), changes in extracellular dopamine, DAT and/or D2 receptors during withdrawal from 10, once daily, 2 mg/kg MA injections. Follow-up biochemical studies conducted in mice selectively bred for high vs. low MA drinking (respectively, MAHDR vs. MALDR mice), provided novel support for anomalies in mesocorticolimbic dopamine as a correlate of genetic vulnerability to high MA intake. Finally, neuropharmacological targeting of NAC dopamine in MA-treated B6 mice demonstrated a bi-directional regulation of MA-induced place-conditioning. These results extend extant literature for MA neurotoxicity by demonstrating that even subchronic exposure to relatively low MA doses are sufficient to elicit relatively long-lasting changes in mesocorticolimbic dopamine and that drug-induced or idiopathic anomalies in mesocorticolimbic dopamine may underpin vulnerability/resiliency to MA addiction.

## Introduction

Methamphetamine (MA) is a potent, highly addictive, amphetamine derivative with intense psychomotor-activating properties. MA abuse is linked to pronounced cognitive, behavioral and emotional deficits, with a high relapse potential, constituting a major public health concern (e.g., Rusyniak, 2011; Dean et al., 2013). While MA ranks second highest as the most commonly abused illicit drug in the world (United Nations Office on Drugs and Crime, 2011), neurobiological research concerning genetic vulnerability to MA abuse/addiction and the impact of early MA experience on the brain to the development of early-stage addiction is limited.

MA is a substrate for plasma membrane monoamine transporters, including the dopamine (DA) transporter (DAT), as well as for the vesicular monoamine transporter, and is reported to also inhibit monoamine oxidase (e.g., Fleckenstein et al., 2007; Chen et al., 2009). Through these mechanisms, MA profoundly increases DA within forebrain terminals, notably nucleus accumbens (NAC), dorsal striatum and prefrontal cortex (PFC) (e.g., Sulzer et al., 2005). As such, the majority of neurobiological research pertaining to MA addiction has focused primarily on MA-forebrain DA interactions (e.g., McCann and Ricaurte, 2004; Yamamoto and Bankson, 2005; Espana and Jones, 2013). The majority of extant pre-clinical data has been derived using very high-dose MA treatment regimens (10–100 mg/kg acutely or binge administration of 4–10 mg/kg given multiple times within a day) that elicit neurotoxicity within dorsal striatal regions (for recent reviews on the subject: Kuhn et al., 2011; Carvalho et al., 2012; Ares-Santos et al., 2013; Halpin et al., 2014). While we have gained tremendous molecular and cellular insight into how high-dose MA experience produces forebrain damage of relevance to late-stage addiction, to the best of our knowledge, less than 15 reports exist pertaining to the interactions between forebrain dopamine systems and low-dose, subchronic exposure to MA (e.g., Zhang et al., 2001; Broom and Yamamoto, 2005; Ago et al., 2006, 2007, 2012; Segal and Kuczenski, 2006; Fukakusa et al., 2008; Schwendt et al., 2009; Lominac et al., 2012; Laćan et al., 2013; Le Cozannet et al., 2013). Also, whereas it is generally held that repeated MA exposure elicits a sensitization of forebrain dopamine release that contributes to the development of behavioral sensitization and/or underpins this drug's positive-reinforcing or rewarding properties (e.g., Ujike et al., 1989; Yang et al., 2008a,b), discrepancies exist regarding the relative roles played by DA within different forebrain terminal regions, most notably the NAC vs. mPFC (see Ago et al., 2006, 2007, 2012).

Moreover, in comparison to the extant data for cocaine and d-amphetamine (c.f., Robinson and Becker, 1986; Vanderschuren and Kalivas, 2000) and for high-dose MA exposure (c.f., Carvalho et al., 2012), we know relatively little regarding how MA exposure early during the use of the drug impacts the brain. As such an understanding has relevance to the transition from MA use to abuse/addiction, the first study presented here examined the effects short and longer-term withdrawal from repeated MA upon basal extracellular DA (DA_EC_) content, DAT and D2/3 receptor (D2/3R) expression, as well as MA-induced DA sensitization, experimenter-administered, MA injections after short-term and longer-term withdrawal in the NAC and PFC. Prior studies have indicated that the effects of contingent vs. non-contingent intravenous MA upon striatal indices of DA_EC_ do not depend highly upon the behavioral contingency of MA delivery and are qualitatively similar (Lominac et al., 2012; Laćan et al., 2013). Thus, we employed a repeated, experimenter-administered injection regimen for this study as this route of MA administration is technically facile in mice.

Another major question pertaining to the neurobiology of MA addiction is why only certain individuals come to repeatedly abuse MA in the first place? This question has also received very little direct experimental attention, until recently (Ikeda et al., 2007; Morita et al., 2008; Phillips et al., 2008; Uhl et al., 2008; Wheeler et al., 2009; Shabani et al., 2011, 2012a,b). In humans, moderate doses of amphetamine-type stimulants (e.g., 0.1–0.4 mg/kg) elicit euphoria and behavioral activation, which are typically considered appetitive/reinforcing; higher, subtoxic, MA doses (e.g., 1.0–4.0 mg/kg) can induce anxiety and elicit psychophysiological symptoms, which can be perceived as aversive (c.f., Cruickshank and Dyer, 2009). As for other drugs of abuse (e.g., Schuckit et al., 1997; Fergusson et al., 2003; Petrakis et al., 2004), individual differences in sensitivity to MA's rewarding vs. aversive effects might influence addiction risk. Thus, experimental attention regarding the neurobiological substrates of MA addiction vulnerability is critical to understand MA addiction etiology, identify potential biomarkers for MA addiction vulnerability/resiliency and develop treatment strategies for early intervention in the disease process. The advent of mice selectively bred to drink higher vs. lower amounts of MA (Methamphetamine High Drinking or MAHDR and Methamphetamine Low Drinking or MALDR) that not only differ in their MA intake and preference under free-access 2-bottle-choice procedures (Wheeler et al., 2009), but also exhibit divergent phenotypes under operant and place-conditioning procedures (Shabani et al., 2011, 2012a,b) provide the opportunity to identify biochemical correlates of high vs. low genetic risk for MA addiction-related behaviors. Thus, we also determined whether or not dopamine anomalies were correlates of genetic vulnerability/resiliency to MA addiction-related behavior and assayed, using neuropharmacological approaches, the role for forebrain DA in MA preference in inbred mice. As the results of an earlier study of MAH/LDR mice suggested anomalies in serotonin (5HT) as a biochemical correlate of high MA intake (Wheeler et al., 2009), we also examined for line differences in indices of forebrain 5HT function.

## Materials and methods

### Subjects

Most studies employed adult, male (8 weeks old) C57BL/6J (B6) mice (Jackson Laboratories, Sacramento, CA). For studies of the biochemical correlates of genetic vulnerability/resiliency for MA intake, adult female MA High Drinking and MA Low Drinking (MAH/LDR) mice on a mixed C57BL/6J and DBA2/J background were generated at the Portland VA Medical Center (see Wheeler et al., 2009) and shipped to UCSB Santa Barbara, where they were acclimatized for a minimum of 6 weeks. Mice were housed in groups under a regular 12-h light:dark cycle (lights off at 19:00 h), with food and water available *ad libitum*. All experimental protocols were consistent with the guidelines put forth by the NIH (NIH Publication No. 80–23, revised 1996) and were approved by the Institutional Animal Care and Use Committees of the University of California Santa Barbara and Oregon Health and Science University.

### Stereotaxic surgery

**Figure 1:**
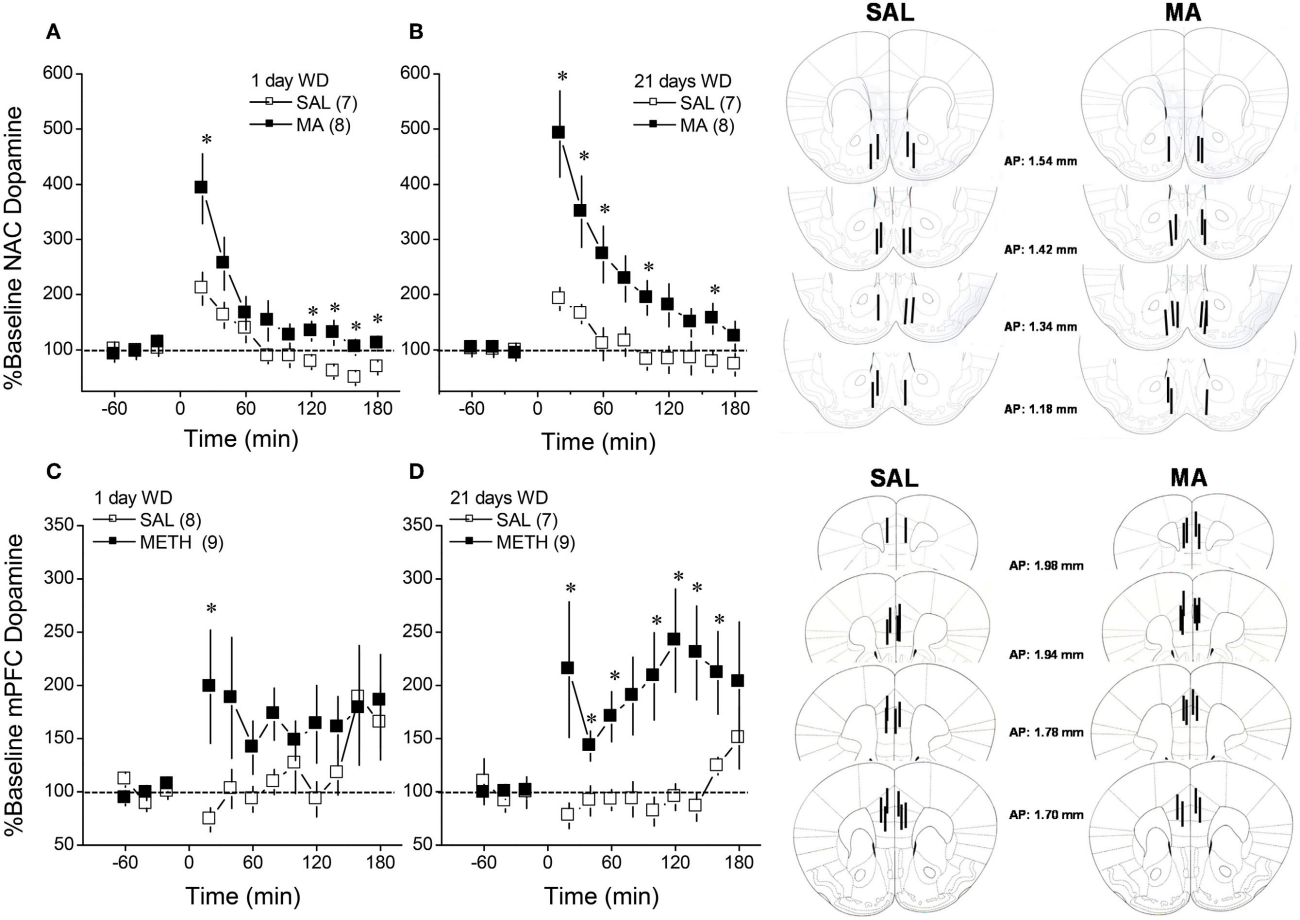
**Summary of the dopamine response to an i.p. challenge injection of 2 mg/kg methamphetamine (MA), administered at either 1 day (left) or 21 days (right) withdrawal (WD), exhibited by B6 mice with a 10-day history of repeated MA (2 mg/kg) or saline (SAL)**. When measured in either the nucleus accumbens (NAC; **A,B**) or the medial prefrontal cortex (mPFC; **C,D**). The data are expressed as the percent change from the average baseline level and represent the means ± s.e.m's of the number of animals indicated in parentheses in each panel. ^*^p < 0.05 vs. SAL (i.e., sensitization).

The surgical procedures for implanting stainless steel guide cannulae (10 mm, 20 gauge, Small Parts; Roanoke, VA) above the mPFC and NAC of mice were identical to those described recently (see Ary et al., 2013). For studies involving *in vivo* microdialysis or microinjection procedures (see below), mice were anesthetized using 1.5–2% isoflurane with 4% oxygen as a carrier gas. Mice were mounted in a stereotaxic device with tooth and ear bars adapted for mice. The animal's skull was exposed, leveled, and holes were drilled based on coordinates from Bregma for the mPFC (AP: +1.8 mm; ML: ± 0.5 mm; DV: −1.0 mm) or NAC (AP: +1.3 mm, ML: ±1 mm, DV: −2.3 mm), according to the mouse brain atlas of Paxinos and Franklin (2007). The guide cannulae were lowered bilaterally such that the tips of the cannulae were 3 mm above the mPFC or border region of the shell and core subregions of the NAC. The skull was then prepared for polymer resin application, the 2 guide cannulae occluded and post-operative care was conducted as described previously (e.g., Ary et al., 2013). Probe placements within the mPFC and NAC were verified prior to any statistical analyses using microscopic analysis of Nissl-stained sections. Only those mice exhibiting correct placement within the boundaries of the mPFC or NAC were included in the statistical analyses of the data (see e.g., Figure 1).

### MA treatment and experimental design

#### Studies of B6 mice

Following either a minimum of 5 days recovery from surgery or following acclimation to the vivarium, B6 mice were randomly assigned to receive either repeated intraperitoneal injections of 2 mg/kg MA (Sigma Aldrich; St Louis, MO) or an equivalent volume of 0.09% saline (SAL; vol = 0.01 ml/kg). MA/SAL injections were administered once daily, for 10 consecutive days, as this regimen is reported to alter NAC DA in rats (Broom and Yamamoto, 2005). *In vivo* microdialysis procedures or sacrifice for immunoblotting were conducted at either 1 or 21 days withdrawal in B6 mice. Whenever possible (see below), the B6 mice underwent 2 identical microdialysis sessions; the first session was conducted at 1 day withdrawal and the second session was conducted at 21 days withdrawal to d and separate groups of animals were used to assay for MA-induced changes in basal DA content, for basal D2R function, for basal DAT function and for MA-stimulated release (*n* = 10–12 at the outset of each assay), as described below.

#### Studies of MAH/LDR mice

Due to the relatively limited number of animals available, the MAH/LDR mice were randomly assigned to *in vivo* microdialysis or immunoblotting studies. Mice in the *in vivo* microdialysis studies were assayed in 2 distinct microdialysis sessions, separated by 2–3 days, and for these sessions, microdialysis probes were lowered into guide cannulae implanted on opposite hemispheres. In one session, we assayed for basal DA or 5HT content using no net-flux procedures (counterbalanced across animals within genotype). In the second session, mice were assayed for the basal content of the other neurotransmitter. Another group of animals only underwent 1 microdialysis session in which we assayed for the effects of an acute injection of MA (2 mg/kg) upon monoamine levels and thus, a microdialysis probe was inserted unilaterally, with the hemisphere counter-balanced across animals. For the immunoblotting studies, half of the MAH/LDR mice were administered an acute injection of 2 mg/kg MA to examine for potential drug effects upon protein expression; the remaining half were administered an acute injection of saline for comparison. At 3 h post-injection, tissue was collected for processing by immunoblotting to be consistent with the experimental design of an earlier report examining for genotype differences in mRNA expression (Wheeler et al., 2009).

#### In vivo microdialysis

Microdialysis was conducted in repeated MA/SAL-treated B6 mice at 1 and 21 days withdrawal or in MAH/LDR mice using procedures very similar to those described recently in Ary et al. (2013). For each microdialysis session, a probe was inserted unilaterally into the mPFC or the NAC, counterbalancing across hemispheres between groups. The animals were then connected a liquid swivel (Instech; Plymouth Meeting, PA) and perfused (2 μ l/min) with microdialysis buffer (NaCl, 147 mM, CaCl_2_, 1.2 mM, KCl, 2.7 mM, MgCl_2_, 1.2 mM, Na_2_HPO_4_, 0.5 mM; adjusted to pH = 7.4). Following a 3-h equilibration period, dialysate collection began and occurred in 20-min intervals into vials containing 10 μ l of preservative (4.76 mM citric acid, 150 mM NaH2PO4, 50 μ M EDTA, 3 mM sodium dodecyl sulfate, 10% methanol (v/v), 15% acetonitrile (v/v), pH = 5.6). The duration of testing ranged from 3–4 h, depending upon the experiment (see below). Upon completion of a session, animals were lightly restrained for probe removal and the dummy cannula reinserted. It should be noted that while each *in vivo* microdialysis study commenced with *n* = 10–12 mice (depending upon the study), we were not always able to obtain data from all animals from the first *in vivo* microdialysis sessions due to a loss of probe patency during sampling, misplaced guide cannula or HPLC malfunction. The final sample sizes indicated in the Results below reflect the final number of animals for which we successfully obtained all samples for the session and for which the probes were localized within the intended region. While not always possible for additional technical reasons (e.g., dislodged cranial implant; clogged cannulae; *n* = 2–4/replicate), a second microdialysis session was conducted by inserting a probe into the guide cannula implanted in the opposite hemisphere. This 2nd session occurred either at 21 days withdrawal from repeated MA/SAL (B6 mice) or a minimum of 3 days following the 1st session (MAH/LDR mice). Thus, of the total number of mice at the outset of each study, we successfully obtained data from both microdialysis sessions for approximately 70% of the subjects tested and this percentage ranged depending upon the study and the number of technical issues that were encountered at the time of sample collection and HPLC analyses.

All microdialysis studies commenced with a 1-h baseline sampling period. To examine the effects of MAH/LDR genotype and of repeated MA treatment on MA-stimulated dopamine release, mice were injected with 2 mg/kg MA and dialysate was collected for an additional 3 h. For the study of repeated MA effects or MAH/LDR genotype differences in basal extracellular dopamine content, we employed quantitative *in vivo* microdialysis procedures, in which increasing concentrations of DA (Sigma Aldrich) were diluted in aCSF to concentrations encapsulating biological levels (0, 2.5, 5, and 10 nM; e.g., Sam and Justice, 1996) and perfused in ascending order for 1 h each. The basal extracellular serotonin (5HT) content was also assayed in MAH/LDR mice (0–10 nM; Sigma Alrich). Linear regression analyses were employed to calculate the point of no net-flux (*y* = 0) and the slope of the plot (i.e., the extraction fraction or Ed; an index of neurotransmitter release and reuptake; e.g., Sam and Justice, 1996), which were analyzed using between-subjects ANOVAs. To relate the MA-induced changes in extracellular dopamine (DA_EC_) observed in B6 mice to drug effects on DAT and D2 autoreceptor function, we assayed the effects of reverse dialyzing the DAT inhibitor GBR-12909 (0, 1, 10, and 100 μ M; Pierce and Kalivas, 1997) or the D2-like DA receptor antagonist (±) sulpiride (0, 50, 100 μ M; Engleman et al., 2004). In the studies of D2-like receptor function, sulpiride was infused *in lieu* of a D2R agonist, as the results of published studies and unpublished pilot studies in our laboratory have failed to reliably detect changes in DA_EC_ using this approach (e.g., Galloway et al., 1986; Santiago et al., 1993; Liu and Steketee, 2011), while the local infusion of D2R antagonists more reliably alter DA_EC_ (Engleman et al., 2004; present study). All compounds were dissolved in microdialysis buffer, although sulpiride required initial dissolution in 50 μ L of acetic acid and the final pH ranged from 6.9–7.3, depending upon the replicate. Each drug concentration was infused for 1 h, akin to the quantitative microdialysis procedures above. Given the known differences in basal DA levels confirmed by no net-flux microdialysis and the susceptibility of these procedures to differences in probe recovery (Westerink and Cremers, 2007), all data for MA-induced neurotransmitter release and for reverse dialysis experiments were expressed as a percent change from baseline. The microdialysis data were analyzed using ANOVAs with repeated measures across the Time or Dose factors and interactions deconstructed for simple effect analyses, followed by *post-hoc* comparisons using *t*-tests.

#### HPLC analysis

Dopamine and serotonin in 27 μl dialysate was measured using high pressure liquid chromatography (HPLC) with electrochemical detection using an ESA Coularray HPLC system (ESA Inc.; Bedford, MA). For this, MD-TM mobile phase was employed (Thermo Dionex), and monoamine neurotransmitters were separated using an MD-150 × 3.2 column (C18, 150 mm × 3.2 mm I.D.; Thermo Dionex). An ESA 5014B analytical cell with two electrodes was used for the electrochemical detection of monoamines—the reduction analytical electrode (E1, −150 mV), and an oxidation analytical electrode (E2, +220 mV). Dopamine and serotonin content in each sample was analyzed by peak height and compared to external standard curves (one for each neurotransmitter) for quantification (e.g., Szumlinski et al., 2007). Unfortunately, our HPLC conditions did not permit reliable detection of norepinephrine in the dialysate.

### Immunoblotting

Immunoblotting was conducted on whole-cell tissue homogenate collected from the mPFC, NAC shell and NAC core of B6 mice at either 1 or 21 days withdrawal from repeated MA treatment (10 × 2 mg/kg) or of MAH/LDR mice 3 h following an acute injection of SAL or 2 mg/kg MA (to be consistent with the experimental design of a prior assay of mRNA levels; Wheeler et al., 2009). To relate MA's effects upon indices of DA_EC_ in B6 and MAH/LDR mice, we examined for the total protein expression of DAT and D2 dopamine receptors (D2Rs), the latter of which serves as an autoreceptor regulating DA_EC_. We also related MA's effect upon indices of 5HT_EC_ in MAH/LDR mice to the total protein expression of SERT and 5HT1B receptors (5HT1BRs), the latter of which serves as an autoreceptor on 5HT terminals. The general procedures used to dissect and homogenize tissue, as well as to separate, transfer and visualize proteins were described recently (Ary et al., 2013). The PFC, NAC shell and NAC core were excised over ice and frozen at −80°C until assay. Tissue was homogenized with a PTFE hand-held tissue grinder in homogenization medium consisting of 0.32 M sucrose, 2 mM EDTA, 1% sodium dodecyl sulfate, 50 μ M phenyl methyl sulfonyl fluoride, and 1 μg/ml leupeptin (pH = 7.2) containing a protease inhibitor (Complete Mini, Roche; Indianapolis, IN) and phosphatase inhibitor cocktail (Sigma). Samples were then subjected to low-speed centrifugation (2000 g). Protein determinations were performed using the Bio-Rad DC protein assay (Bio-Rad; Hercules, CA). Samples (30 μ g total protein) were subjected to a sodium dodecyl sulfate-polyacrylamide gradient gel (4–12% Bis-Tris or 3–8% Tris-Acetate Invitrogen; Carlsbad, CA) electrophoresis, transferred via standard apparatus (Bio-Rad) to nitrocellulose membrane. Depending upon the study, the total protein content of DAT, D2Rs, SERT, and 5-HT1BRs were determined by immunoprobing using the following rabbit polyclonal antibodies: anti-D2R (1:333–1:500, Novus Biologicals), anti-DAT (1:333–1:1:500, Millipore), anti-SERT (1:500, Millipore), anti-5HT1BR (1:250–1:500; Lifespan Biosciences). Anti-Calnexin (1:1000, Millipore) was used to control for protein loading and to normalize the expression of the protein of interest. Immuno-labeled proteins were detected using horseradish peroxidase-conjugated secondary IgGs (diluted 1:20,000–1:80,000) (Jackson Immuno Research) and visualized with enhanced chemiluminescence (Amersham Life Sciences). Immunoreactive levels were quantified by integrating band density × area using computer-assisted densitometry (NIH ImageJ version 1.60). The density × area measurements were averaged over 3–4 control samples for each gel and all bands were expressed as percent of the control values (SAL-1 day withdrawal for the repeated MA study; SAL-F2B6D2 mice for the genetic study). The immunoblotting data were analyzed using ANOVAs, followed by analyses for main effects and *t*-tests for *post-hoc* comparisons, when appropriate.

### MA-induced place-conditioning

Groups of B6 mice were implanted with guide cannulae above the mPFC or NAC and then subjected to a MA place-conditioning regimen that was similar to that employed previously for stimulants in our laboratory (Ary et al., 2013). The study proceeded in five sequential phases: habituation, pre-conditioning test (Pre-Test), MA/SAL conditioning, post-conditioning test (Post-Test), and microinjection test (Microinjection Test). All sessions were 15 min in duration and animals received no systemic injections during the habituation, Pre-Test, Post-Test or Microinjection Test, when they had free-access to both compartments of the apparatus. For conditioning, mice received 4 alternating pairings of distinct compartments with either MA (2 mg/kg; vol = 0.01 ml/kg) or an equivalent volume of saline in an unbiased fashion. One compartment had black and white marble-patterned walls, with a textured floor, while the other compartment had wood-patterned walls, with a smooth Plexiglas floor. The difference in the time spent in the drug-paired vs. unpaired compartment (CPP Score) on the Post-Test served to index place-conditioning prior to any intracranial manipulation. Having established that MA elicited a conditioned place-preference (CPP), mice were assigned to receive an intracranial infusion (0.5 μl/side) of 100 nM GBR-12909, 100 nM of the D2/3R agonist quinpirole or an aCSF vehicle. The doses of GBR-12909 and quinpirole were selected to be maximally effective for raising and lowering, respectively, extracellular dopamine in on-going microdialysis studies in our laboratory. Microinfusions were delivered at a rate of 0.5 ul/min via 33-gauge stainless steel tubing (12 mm in length). Microinjectors were left in place for an additional 1 min prior to careful removal. Five min later, mice were placed into the place-conditioning apparatus for 15 min. Following testing, microinjector placements within the mPFC or NAC were verified in Nissl-stained coronal tissue sections (Figure 5). The data were analyzed using ANOVAs.

## Results

### DA sensitization in MA-treated B6 mice

We characterized the short- and longer-term effects of a history of subchronic MA upon MA-induced DA release within NAC and mPFC in B6 mice. As illustrated in Figure 1, acute MA (2 mg/kg) elicited a rise in extracellular dopamine that exhibited a clear sensitization in MA-treated animals (Figure 1; compare open vs. closed symbols in each panel) [for NAC, Repeated Treatment × Time: *F*_(11, 297)_ = 4.40, *p* < 0.0001; for mPFC: Repeated Treatment × Time: *F*_(11, 319)_ = 2.06, *p* = 0.02]. Although the magnitude and time-courses of this effect varied between regions, repeated MA sensitized DA release in both regions in a time-independent fashion as indicated by no main or interaction effects of the Withdrawal factor for either region (*p*'s > 0.20).

### Basal DA content in MA-treated B6 mice

**Figure 2:**
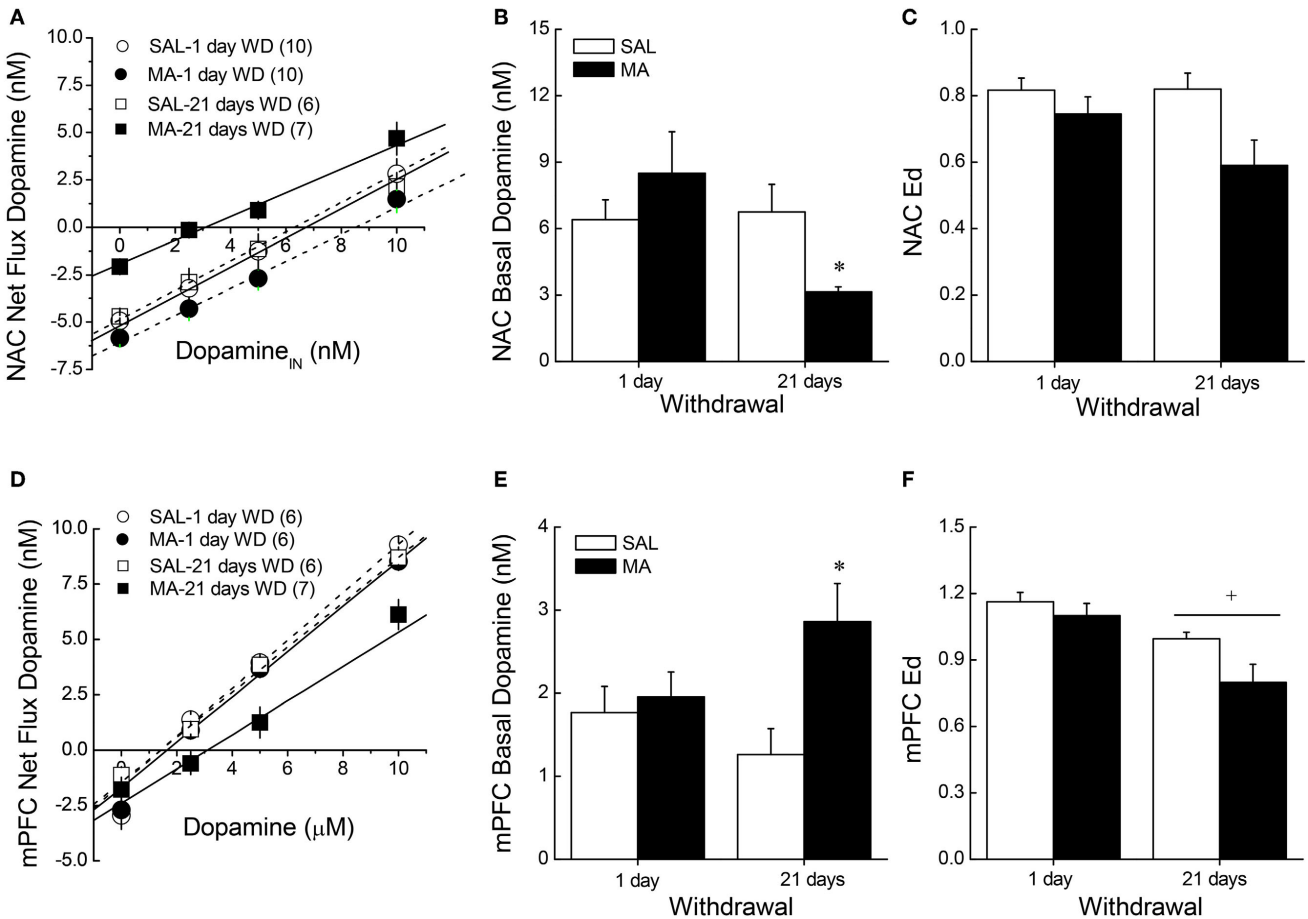
**Summary of the results of a dopamine no net-flux *in vivo* microdialysis study conducted at either 1 or 21 days withdrawal (WD) in B6 mice with a 10-day history of repeated methamphetamine (2 mg/kg; MA) or saline (SAL) within the NAC core-shell interface (A–C) and the mPFC (D–F)**. Linear regression analyses **(A,D)** conducted on the plots of the net flux of dopamine vs. the amount of dopamine infused revealed time-dependent changes in *y* = 0 **(B,E)**, an estimate of basal extracellular dopamine content, in MA-sensitized mice within both the NAC and the mPFC. **(C,F)** There were no group differences in the slopes/extraction fractions (Ed) of the linear regressions. The data represent the means ± s.e.m's of the number of animals indicated in parentheses in each panel. ^*^p < 0.05 vs. SAL; ^+^*p* < 0.05 vs. 1-day WD.

When obtained by conventional microdialysis, the average basal DA_EC_ levels within both the NAC and mPFC were moderately lower in MA-treated mice at 21 days withdrawal (Table 1). However, statistical analyses of the data failed to identify any significant main or interaction effects (all *p*'s > 0.06). As the results from conventional microdialysis procedures are subject to differences in probe recovery, we then employed quantitative microdialysis procedures to examine the data for MA-induced changes in basal DA_EC_ and DA clearance from the probe. Using no net-flux procedures and linear regression analyses (Figures 2A,D), we observed time-dependent changes in basal DA_EC_ content (*y* = 0) in MA-sensitized mice within both the NAC (Figure 2B) [Treatment × Withdrawal: *F*_(1, 29)_ = 4.162, *p* = 0.05] and the mPFC (Figure 2E) [Treatment × Withdrawal: *F*_(1, 21)_ = 4.767, *p* = 0.04]. SAL-MA differences in *y* = 0 were not present in either region at 1 day withdrawal (*t*-tests, p's > 0.05), but group differences were apparent at 21 days withdrawal [for NAC, *t*_(11)_ = 3.08, *p* = 0.01; for mPFC, *t*_(11)_ = 3.13, *p* = 0.01]. The MA-induced reduction in DA_EC_ content was unrelated to changes in release/reuptake, as group differences were not observed regarding Ed derived from the slopes of the linear regressions for either region (Figures 2C,F) [for NAC, Treatment effect: *F*_(1, 29)_ = 7.48, *p* = 0.01; no Withdrawal main or interaction effect, p's > 0.15; for mPFC, Treatment: *F*_(1, 21)_ = 4.81, *p* = 0.04; Withdrawal: *F*_(1, 21)_ = 15.831, *p* = 0.001; interaction: *p* = 0.27].

**Table 1:** **When assayed using conventional *in vivo* microdialysis procedures, we failed to detect an effect of repeated methamphetamine experience or withdrawal upon basal extracellular dopamine (fg/20 μl dialysate) within either the mPFC or the NAC**.

	**Saline**		**Methamphetamine**	
	**1 day WD**	**21 days WD**	**1 day WD**	**21 days WD**
mPFC	0.55 ± 0.13 (8)	0.78 ± 0.16 (7)	0.95 ± 0.25 (9)	0.59 ± 0.16 (9)
NAC	3.60 ± 0.74 (7)	3.78 ± 0.92 (7)	3.03 ± 1.22 (8)	1.00 ± 0.24 (8)

*The data represent the mean ± s.e.m. of the number of animals indicated in parentheses*.

### DAT expression and function in MA-treated B6 mice

**Figure 3:**
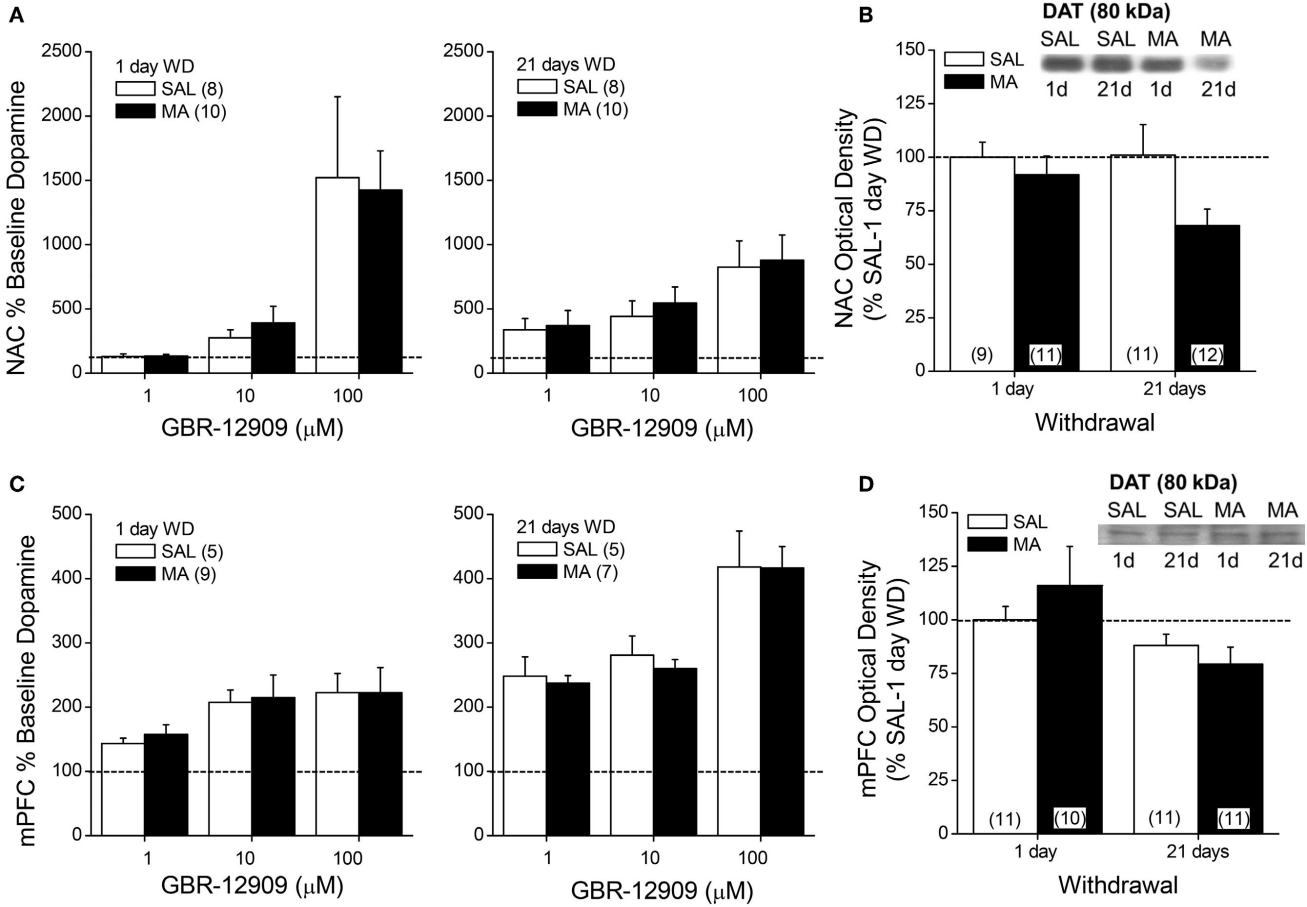
**Summary of the effects of a 10-day history of repeated methamphetamine (MA; 2 mg/kg) or saline (SAL) upon the capacity of the DAT reuptake inhibitor GBR-12909 to elevate extracellular dopamine (A,C) and upon total DAT protein expression (B,D) within the NAC (top panels) and mPFC (bottom panels) at 1 and 21 days withdrawal (WD)**. The data are presented as the percent change from the average baseline level of neurotransmitter and represent the means ± s.e.m's of the number of animals indicated in parentheses in each panel.

We next related MA-induced changes in DA_EC_ to the expression and function of DAT using microdialysis and immunoblotting approaches. MA withdrawal did not affect the capacity of GBR-12909 to elevate DA_EC_ levels within either the NAC (Figure 3A) or the mPFC (Figure 3C) (for both regions, Dose effects: *p*'s < 0.001; no main or interaction effect of the Repeated Treatment factor: *p*'s > 0.05). MA treatment also did not alter DAT expression within the NAC shell (not shown) or mPFC (Figure 3D; Two-Way ANOVA's, *p*'s > 0.05). However, drug treatment reduced DAT expression within the NAC core (Figure 3B), although the results did not support a time-dependency to this effect [Repeated Treatment effect: *F*_(1, 39)_ = 4.26, *p* = 0.05; no Withdrawal effect or interaction, *p* > 0.20].

### D2R expression and function in MA-treated B6 mice

**Figure 4:**
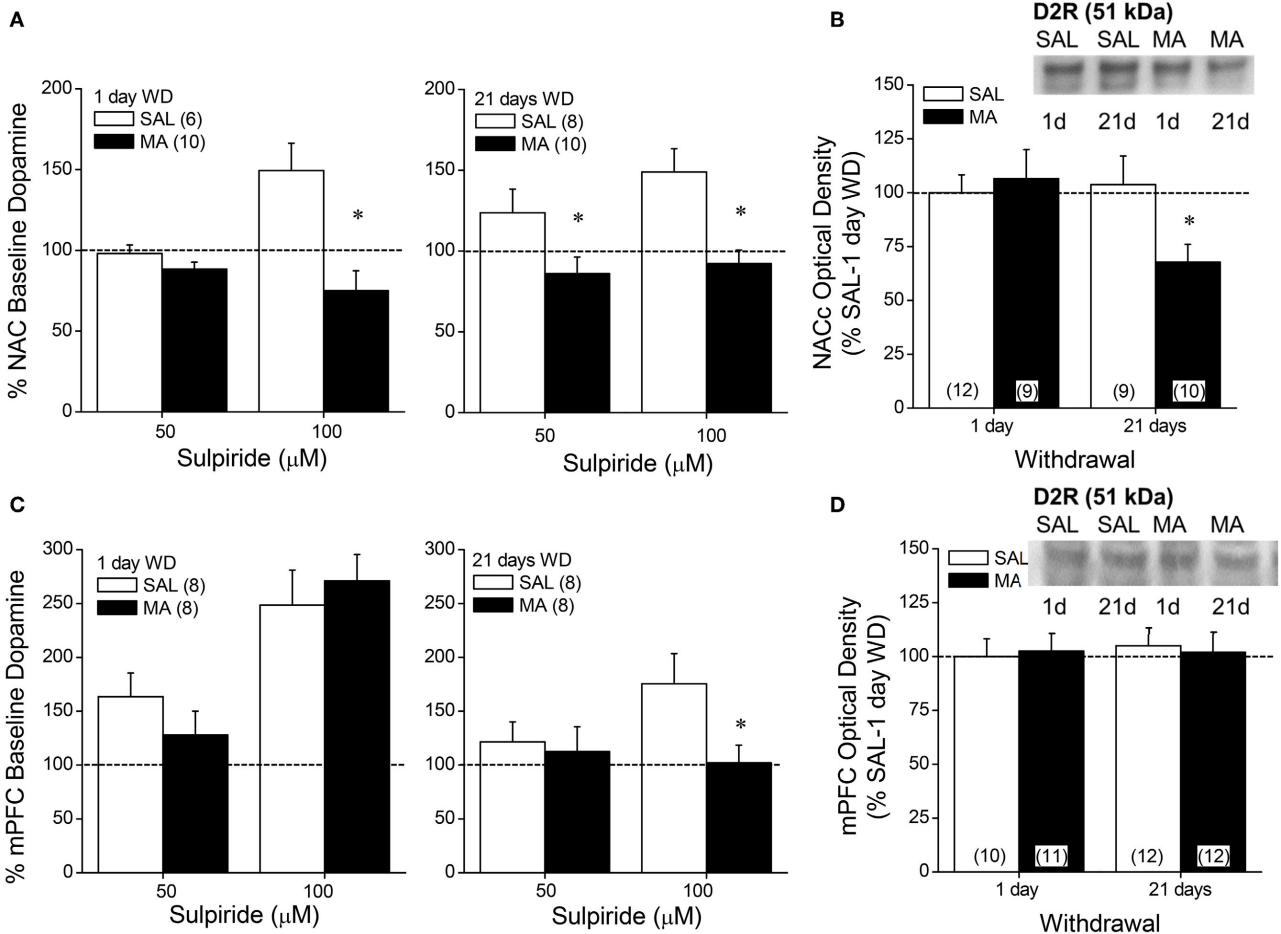
**Summary of the effects of a 10-day history of repeated methamphetamine (MA; 2 mg/kg) or saline (SAL) upon the capacity of the D2/D3 receptor antagonist sulpiride to elevate extracellular dopamine (A,C) and upon total D2 receptor protein expression (B,D) within the NAC (top panels) and mPFC (bottom panels) at 1 and 21 days withdrawal (WD)**. The data are presented as a percent change from the average baseline dopamine levels and represent the means ± s.e.m's of the number of animals indicated in parentheses in each panel. ^*^p < 0.05 vs. respective SAL.

We then related MA-induced changes in DA_EC_ to the expression and function of D2/3Rs using microdialysis and immunoblotting approaches. MA withdrawal blunted D2/3R function within the NAC at both withdrawal time-points (Figure 4A) [1 day: Dose × Repeated Treatment: *F*_(1, 14)_ = 11.18, *p* = 0.005; *t*-tests; 21 days: effects of Dose and Treatment: *p*'s < 0.006, but no interaction: *p* = 0.27]. MA animals also exhibited reduced receptor expression at the 21-day withdrawal time-point within the NAC core (Figure 4B) [Repeated Treatment × Withdrawal (α = 0.1 based on microdialysis results): *F*_(1, 38)_ = 3.739, *p* = 0.06; *post-hoc*, 1 day WD, Repeated Treatment: *p* = 0.68; 21 days WD, *F*_(1, 18)_ = 5.378, *p* = 0.03]. No change in D2R expression was observed within the NAC shell (Two-Way ANOVAs, α = 0.1, all *p*'s > 0.25). While MA history did not impact the sulpiride-induced rise in DA within the mPFC at 1-day withdrawal [Dose effect: *F*_(1, 13)_ = 19.12, *p* < 0.001; no main or interaction effect of the Repeated Treatment factor, *p*'s > 0.10], MA reduced responsiveness to the 100 μM dose in the long-term (Figure 4C) [Dose × Repeated Treatment: *F*_(1, 14)_ = 8.56, *p* = 0.01; *t*-tests: *p*'s < 0.05]. However, we failed to detect changes in D2R expression within the mPFC (Figure 4D; Two-Way ANOVA's, all *p*'s > 0.05).

### MA-induced CPP in B6 mice

**Figure 5:**
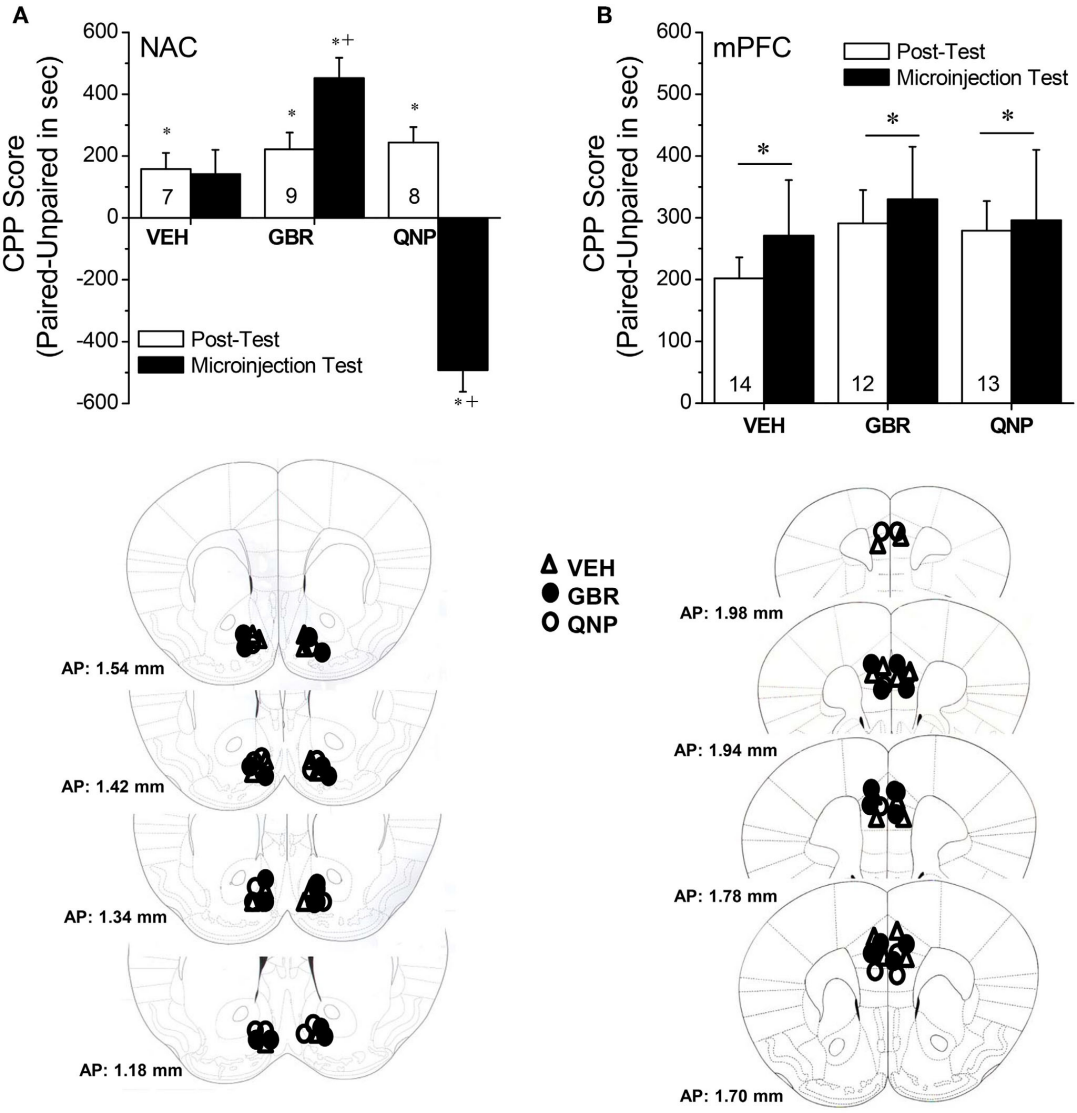
**Summary of changes in the difference in the time spent (in sec) in an environment previously paired with 2 mg/kg methamphetamine (Paired) vs. an environment paired previously with saline (Unpaired) (i.e., CPP Score) produced by intra-NAC (A) or intra-mPFC (B) infusions of 100 nM of the DAT reuptake inhibitor GBR-12909 (GBR) or 100 nM of the D2/3 receptor agonist quinpirole (QNP)**. The data represent the means ± s.e.m's of the number of animals indicated in parentheses in each panel. ^*^denotes *p* < 0.05 Paired vs. Unpaired; ^+^*p* < 0.05 vs. respective VEH.

The data presented above indicated that subchronic MA exposure was sufficient to produce enduring anomalies in DA_EC_ within both the mPFC and NAC. As the role for DA_EC_ in mediating MA preference has not been fully vetted, we examined the effects of raising (via site-directed infusions of the DAT inhibitor GBR-12909) or lowering (via site-directed infusions of the D2/3 autoreceptor agonist quinpirole) DA_EC_ upon the expression of a MA-conditioned place-preference. Neuropharmacological manipulation of the NAC impacted the expression of a MA-induced place-conditioning in B6 mice (Figure 5A) [Side × Test × Drug: *F*_(2, 21)_ = 18.60, *p* < 0.0001]. In the absence of intra-NAC infusion (Post-test), there were no group differences in CPP magnitude (Side × Treatment, *p* > 0.50). However, group differences emerged with respect to both the extent and direction of place-conditioning upon intra-NAC microinjection [Side × Treatment: *F*_(2, 21)_ = 48.49, *p* < 0.0001]. Vehicle-infused animals exhibited a non-significant CPP [Side effect: *p* = 0.11], GBR12909 facilitated CPP expression [Side effect: *F*_(1, 8)_ = 49.00, *p* < 0.0001] and quinpirole elicited a marked conditioned place-aversion (CPA) [Side effect: *F*_(1, 7)_ = 48.52, *p* < 0.0001]. An analysis of CPP scores for the Microinjection Test confirmed greater CPP in GBR12909-infused animals vs. vehicle controls [*F*_(2, 21)_ = 48.49, *p* < 0.0001; *t*-tests, *p*'s < 0.05]. In contrast, intra-mPFC DA manipulations failed to alter CPP expression across 2 replicates of study (Figure 5B) [Side effect: *F*_(1, 39)_ = 32.72, *p* < 0.0001; Test effect: *F*_(1, 39)_ = 11.85, *p* < 0.0001; no main or interaction effect of the Drug factor, *p*'s > 0.30].

### Monoamine content in MAH/LDR mice

**Figure 6:**
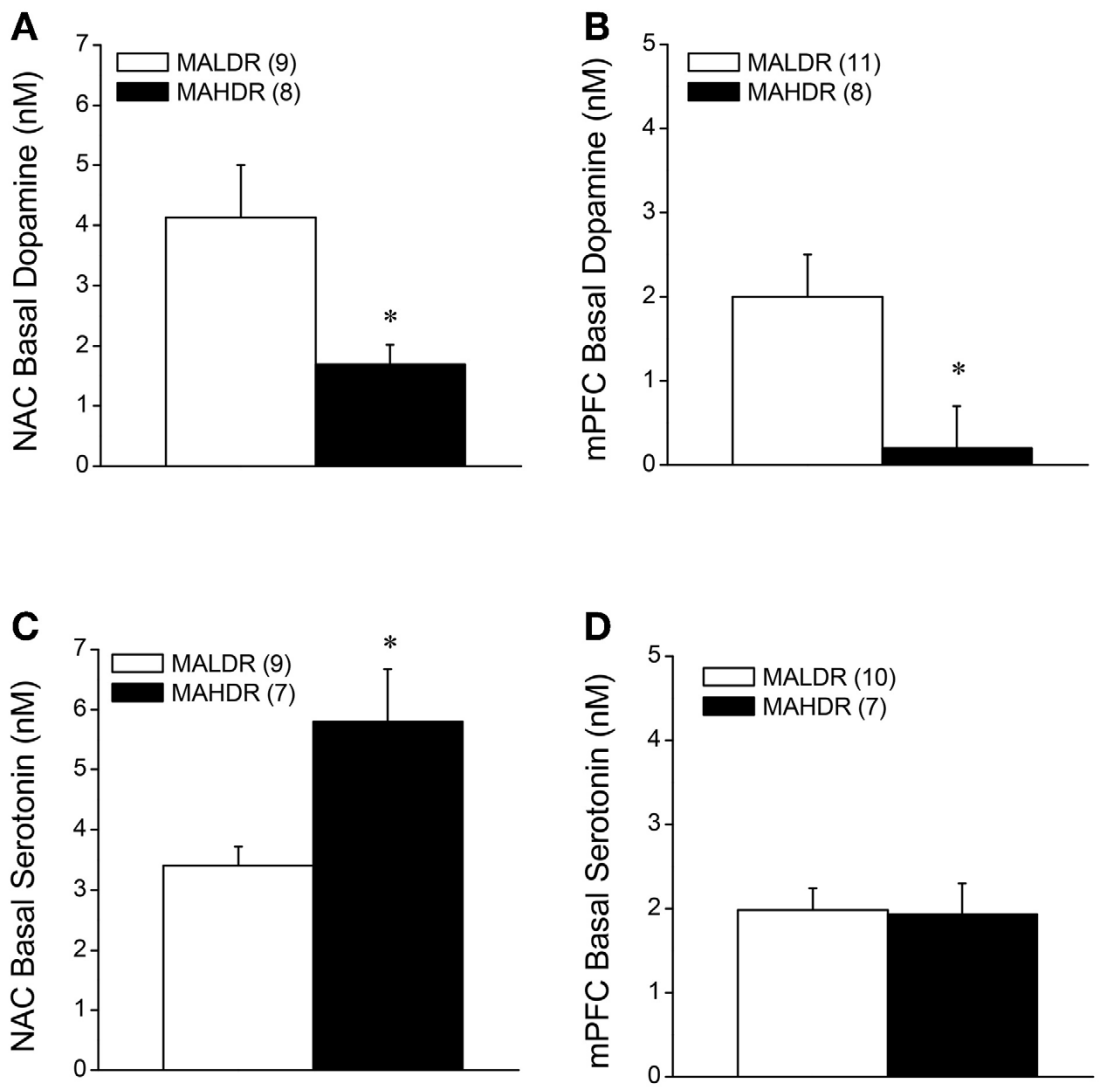
**Summary of the results of a dopamine (A,B) and serotonin (C,D) no net-flux *in vivo* microdialysis study conducted within the NAC core-shell interface (left) and mPFC (right) in MA-naïve mice on a genetically heterogeneous B6 × D2 background (F2B6D2) and mice selectively bred for high vs. low MA intake (MAHDR and MALDR, respectively)**. The data represent the means ± s.e.m's of the number of animals indicated in parentheses in each panel. ^*^p < 0.05 vs. MALDR.

The data for MA-injected B6 mice indicate that a history of subchronic MA exposure is sufficient to produce enduring alterations in basal DA_EC_ within both the NAC and mPFC (Figure 2). Moreover, the neuropharmacological results supported an important role for DA_EC_, particularly within the NAC, in mediating MA-conditioned preference and aversion (Figure 5). Thus, we determined whether or not the divergent behavioral phenotypes of MAH/LDR mice might relate to differences in basal DA_EC_ content. We also examined for differences in basal 5HT_EC_ content, as a prior examination for biochemical correlates of genetic vulnerability to high MA intake indicated higher expression of the mRNA encoding the serotonin transporter SERT within the NAC of MAHDR vs. MALDR mice (Wheeler et al., 2009). Using no net-flux approaches, MAHDR-MALDR differences were noted for NAC basal DA_EC_ content (Figure 6A) [*t*_(15)_ = 2.50, *p* = 0.02], with levels being lower in MAHDR vs. MALDR mice. mPFC DA_EC_ content also varied with genotype (Figure 6B) [*t*_(15)_ = 2.41, *p* = 0.03] and again, MAHDR animals exhibited lower DA content. While no genotypic differences were noted for mPFC 5HT_EC_ content (Figure 6D; *t*-test, *p* = 0.92), NAC 5HT_EC_ content varied with genotype (Figure 6C) [*t*_(14)_ = 2.89, *p* = 0.01], with MAHDR mice exhibiting higher serotonin levels than MALDR animals. No genotypic differences were observed for the Ed for either DA or 5HT within either brain region (Table 2; One-Way ANOVA's, all *p*'s > 0.35). Thus, the genotypic differences in extracellular neurotransmitter content were not obviously related to neurotransmitter clearance/release.

**Table 2:** ***In vivo* extraction fractions (*E_d_*) for dopamine and serotonin in the medial prefrontal cortex (mPFC) and the nucleus accumbens core-shell interface (NAC) of mice selectively bred for high vs. low methamphetamine drinking (respectively, MAHDR, and MALDR), as determined using quantitative microdialysis approaches**.

	**MAHDR**	**MALDR**
NAC dopamine	0.96±0.03 (8)	0.89±0.07 (9)
NAC serotonin	1.00±0.15 (7)	1.05±0.09 (9)
mPFC dopamine	0.85±0.14 (8)	0.84±0.07 (11)
mPFC serotonin	1.41±0.23 (8)	1.49±0.22 (11)

*The data represent the mean ± s.e.m. of the number of animals indicated in parentheses*.

### MA-stimulated monoamine release in MAH/LDR mice

The results of the quantitative microdialysis studies indicated line differences for basal DA_EC_ and 5HT_EC_ content (Figure 6). Thus, we examined also for line differences in the capacity of an acute MA injection (2 mg/kg) to elevate DA and 5HT levels within the NAC and mPFC. A summary of the average baseline levels of DA and 5HT within the NAC and mPFC is provided in Table 3. As the absolute amount of neurotransmitter detected by conventional microdialysis procedures is subject to influences by individual probe recovery (Westerink and Cremers, 2007), the results obtained under conventional microdialysis procedures did not match exactly those obtained under quantitative microdialysis procedures. Notably, our conventional microdialysis procedures did not detect line differences in basal NAC neurotransmitter levels (*t*-tests, *p*'s > 0.10). However, we did detect lower DA_EC_ and higher 5HT_EC_ within the mPFC of MAHDR vs. MALDR mice [for DA, *t*_(14)_ = 2.18, *p* = 0.04; for 5HT, *t*_(14)_ = 3.63, *p* = 0.003].

**Table 3:** **Basal extracellular levels of dopamine and serotonin (in fg/27 μl sample) within the medial prefrontal cortex (mPFC) and the nucleus accumbens (NAC) of mice selectively bred for high vs. low methamphetamine drinking (MAHDR and MALDR, respectively), as determined using conventional microdialysis approaches**.

	**MAHDR**	**MALDR**
NAC dopamine	8.75±1.13 (8)	7.82±1.26 (9)
NAC serotonin	3.99±0.74 (9)	3.42±0.59 (7)
mPFC dopamine	1.38±0.33^*^(7)	2.78±0.64 (9)
mPFC serotonin	6.96±0.80^*^(7)	3.32±0.66 (9)

*The data represent the mean ± s.e.m. of 3 dialysate samples, collected during the hour prior to a methamphetamine injection. The number of animals employed in the statistical analyses of the data is indicated in parentheses*.

* **p* < 0.05 (t-test)*.

**Figure 7:**
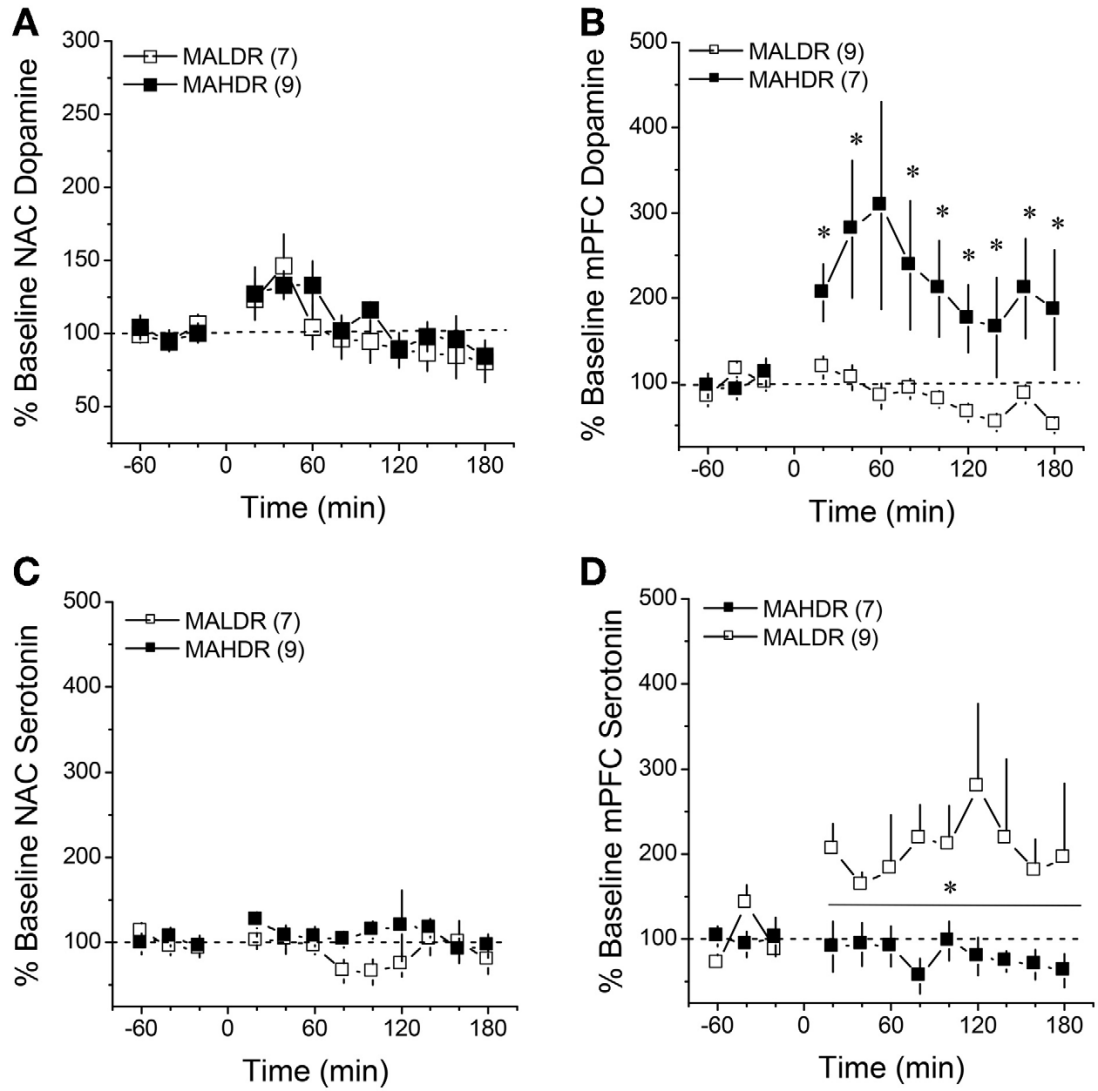
**Summary of the change in extracellular dopamine (A,B) and serotonin (C,D) exhibited within the NAC core-shell interface (left) and mPFC (right) of genetically heterogeneous B6 × D2 mice (F2B6D2) and mice selectively bred for high vs. low MA intake (MAHDR and MALDR, respectively) administered an acute injection of 2 mg/kg MA**. The data are expressed as a percent change from the average baseline values and represent the means ± s.e.m's of the number of animals indicated in parentheses in each panel. ^*^p < 0.05 vs. average baseline.

Surprisingly, no line differences were observed for MA-stimulated DA release within the NAC (Figure 7A) [Time effect: *F*_(11, 154)_ = 5.09, *p* < 0.0001; interaction: *p* = 0.85]. In contrast, marked differences were observed for MA-induced DA release in the mPFC (Figure 7B) [Genotype × Time: *F*_(22, 154)_ = 3.12, *p* = 0.001]. As illustrated in Figure 7B, 2 mg/kg MA injection produced a robust (2–3-fold increase) in mPFC DA levels in MAHDR mice [F_(11, 66)_ = 2.33, *p* = 0.02]. In contrast, the MA injection did not elevate mPFC DA levels at all in MALDR mice; rather, their DA levels dropped below baseline post-injection [*F*_(11, 88)_ = 4.82, *p* < 0.0001]. Acute MA failed to alter 5HT levels in the NAC of either genotype (Figure 7C; Two-Way ANOVA, all *p*'s > 0.05). However, there was an overall genotypic difference for MA-induced increases in mPFC 5HT (Figure 7D) [Genotype effect: *F*_(1, 14)_ = 5.49, *p* = 0.03; no main or interaction effects of Time: *p*'s > 0.20], with MALDR mice exhibiting a 2 to 2.5-fold elevation in mPFC 5HT levels post-injection that persisted throughout the microdialysis session and MAHDR mice exhibiting no sign of MA-induced 5HT release (Figure 7D).

### Immunoblotting in MAH/LDR mice

**Figure 8:**
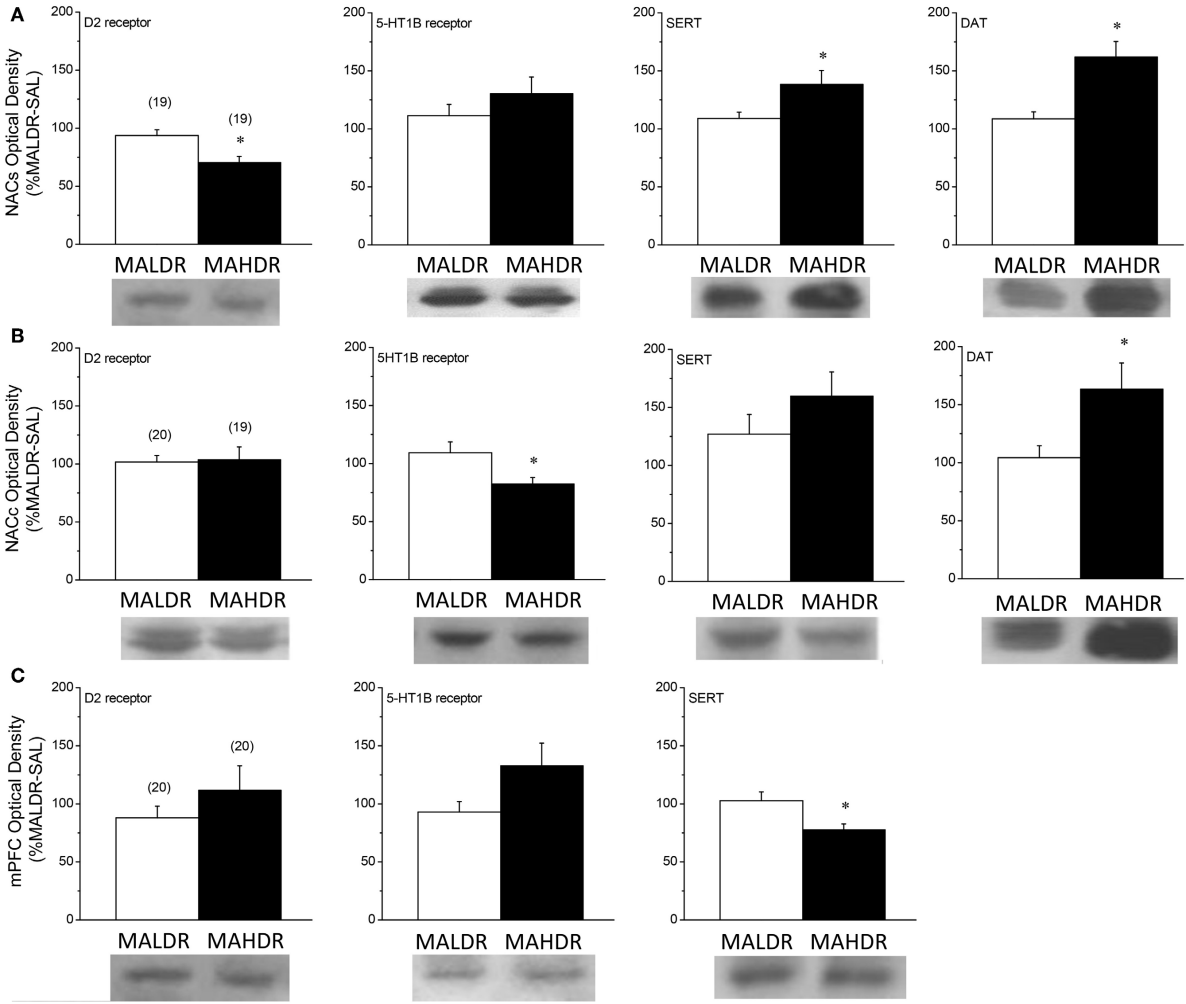
**Comparison of protein expression within the nucleus accumbens shell (NACs) (A), the nucleus accumbens core (NACc) (B) and the medial prefrontal cortex (mPFC) (C) for the D2 dopamine receptor, the dopamine transporter (DAT), the 5HT1B receptor and the serotonin transporter (SERT) of genetically heterogeneous B6 × D2 mice (F2B6D2) and mice selectively bred for high vs. low MA intake (MAHDR and MALDR, respectively)**. As the results of the statistical analyses of the data for all proteins failed to indicate an effect of the MA challenge injection upon protein expression, the data were collapsed across injection groups for clarity of presentation of genotypic differences. The data represent the means ± s.e.m's of the number of animals indicated in parentheses in each panel. ^*^p < 0.05 vs. MALDR.

Given genotype differences in basal and MA-stimulated neurotransmitter release (Figures 6, 7), we next employed immunoblotting to index the expression of DAT, SERT, D2R, and 5HT1BR in the selected lines (Figure 8). The results of the statistical analyses for all proteins failed to indicate any main or interaction effects of the Treatment factor (all *p*'s > 0.05). Thus, the data were collapsed across treatments for clarity of presentation. In the NAC shell (Figure 8A), we observed genotypic differences in D2R [Genotype effect: *F*_(1, 37)_ = 10.63, *p* = 0.003], DAT [Genotype effect: *F*_(1, 37)_ = 13.53, *p* = 0.001] and SERT [Genotype effect: *F*_(1, 37)_ = 4.90, *p* = 0.03] expression, with MAHDR mice exhibiting lower D2R levels, but higher transporter levels, vs. MALDR animals. Genotypic differences were not observed for NAC shell 5HT1BR expression. In contrast MAHDR mice exhibited higher DAT and SERT levels relative to MALDRalso varied with genotype within the NAC shell, but this effect did not reach statistical significance [Genotype effect: *p* = 0.06]. SERT levels varied significantly with genotype in the NAC shell [Genotype effect: *F*_(2, 57)_ = 4.79, *p* = 0.01], with MAHDR mice exhibiting higher expression vs. the other genotypes (LSD *post-hoc* tests, *p*'s < 0.02). No genotypic difference in 5HT1BR was observed in the NAC shell (Genotype effect, *p* > 0.35). In the NAC core (Figure 8B), MAHDR mice exhibited lower 5HT1BR expression but higher DAT expression than MALDR animals [for 5HT1BR, Genotype effect: *F*_(1, 38)_ = 6.13, *p* = 0.02; for DAT, Genotype effect: *F*_(1, 38)_ = 5.46, *p* = 0.03]. Genotypic differences were not noted for D2R or for SERT expression within the NAC core (Genotype effects, *p*'s > 0.10). In the mPFC (Figure 8C), we observed no line differences for the D2R (Genotype effect: *p* = 0.29). Overall, MAHDR exhibited higher 5HT1B levels than MALDR mice, but this difference was shy of statistical significance (Genotype effect: *p* = 0.07). and the rise in 5HT1BR expression observed in MAHDR. there was a moderate genotypic difference in 5HT1BRs that reflected lower levels in both selected lines, compared to F2B6D2 mice [Genotype effect: *F*_(2, 59)_ = 2.99, *p* = 0.06]. A similar pattern of genotypic differences were observed for the D2 receptor [Genotype effect: *F*_(2, 59)_ = 4.68, *p* = 0.01], with F2B6D2 mice exhibiting significantly higher receptor levels, compared to both selected lines (LSD *post-hoc* tests, *p*'s < 0.05). We could not detect DAT within the mPFC of the mice in this study, and no differences were noted for SERT expression (Genotype effect: *p* > 0.45).

## Discussion

An understanding of the neurobiological substrates of MA addiction vulnerability and the effects of subchronic, subtoxic, MA experience upon the brain is critical to understanding the etiology of MA addiction, identifying potential biomarkers for MA addiction vulnerability/resiliency and developing treatment strategies for early intervention in the disease process. The present studies were conducted to further extend knowledge regarding the interactions between subchronic MA exposure and forebrain DA and to relate anomalies in forebrain DA and 5HT to genetic vulnerability/resiliency to high MA intake.

### Subchronic MA elicits a time-independent sensitization of corticolimbic DA

Regional differences existed in MA's ability to elicit DA release within the 2 major terminal regions of the mesocorticolimbic DA system and regional differences were apparent with respect to the time-course of DA release in both acute and repeated MA-treated animals (Figure 1). As reported previously (Shoblock et al., 2003), MA elicited a markedly larger rise in DA_EC_ within the NAC than within the mPFC, although the MA-induced elevation in DA_EC_ produced within the mPFC was more persistent than that observed within the NAC. This regional distinction in the DA response to MA might relate to regional differences in DAT expression, which is higher in striatal vs. frontal cortical structures (Sesack et al., 1998). Higher DAT expression within the NAC could account for the larger magnitude of MA effect and the faster rate of decline in DA_EC_ observed in the NAC, relative to the mPFC. Regional differences exist also in the relative roles played by metabolizing enzymes, monoamine oxidase (MAOs) and catechol-O-methyltransferase (COMT), in determining DA_EC_ (e.g., Karoum et al., 1994). MAO can be inhibited by MA (e.g., Fleckenstein et al., 2007; Chen et al., 2009) and by virtue of the fact that the majority of DA released within striatal structures is removed into neuron terminals by DAT (Sesack et al., 1998), MAOs play a more critical role in regulating DA_EC_ within striatum than they do within frontal cortex, where DAT expression is relatively low (Karoum et al., 1994). However, the fact that the MA-induced rise in DA_EC_ was less persistent within NAC than within mPFC (Figure 1), argues less in favor of a role for MA inhibition of MAO as a major contributing factor to the rise in NAC DA observed in acute MA-injected animals.

Regardless of regional differences in the time-course of MA-stimulated DA release, a subchronic history of MA was sufficient to elicit DA sensitization within both the NAC and the mPFC of male B6 mice. It is notable that in both the cases of the NAC and the mPFC, two features of the time-course of MA-induced DA release varied as a function of MA experience: the magnitude of the initial rise and the persistence of the rise, particularly during the last hour post-injection. Markedly apparent for both regions, MA-experienced animals exhibited a higher initial rise in DA_EC_ post-injection than did animals acutely administered drug. Such findings suggest the repeated MA experience may increase the amount or function of plasma membrane or vesicular transporters or in the availability of vesicular DA for release. While we did not assay for changes in the levels of the vesicular transporter, we did not detect any obvious relation between sensitized DA release and the protein levels of DAT within either the NAC or mPFC. Moreover, we failed to detect SAL-MA differences in the rise in DA_EC_ produced by infusion of GBR-12909, which depends upon both the availability of DAT for binding and the integrity of impulse-dependent DA release mechanisms. Thus, at the present time, it would not appear that alterations in DAT function/expression or in mechanisms regulating the releasability of DA contribute significantly to the sensitization of the initial rise in DA_EC_ observed in MA-experienced mice, although these mechansims were cannot be vetted thoroughly using *in vivo* microdialysis and conventional immunoblotting methods. The fact that the rise in DA_EC_ elicited by the MA challenge injection was more persistent in MA-experienced vs. acutely treated animals (Figure 1) suggests that perhaps repeated drug experience lowered DA catabolism. As mentioned above, MA inhibits MAO (Fleckenstein et al., 2007) and the possibility exists that with repeated drug experience, this mechanism may contribute to this drug's capacity to promote higher DA_EC_ levels, particularly within the NAC (Popova et al., 2004). In contrast to striatum, frontal cortical structures exhibit low DAT expression (Sesack et al., 1998). As such, DAT and MAOs play less of a role in DA catabolism within frontal cortex than they do within subcortical regions (e.g., Karoum et al., 1994) and DA catabolism is mediated in large part by COMT, particularly under conditions of elevated DA_EC_ such as those produced by MA treatment (e.g., Karoum et al., 1994; Huotari et al., 2002a,b; Matsumoto et al., 2003). While speculative at this time, the possibility exists that repeated MA treatment reduces also the function of COMT, via some indirect mechanism, that may promote the amount of DA_EC_. Given the reliance of mPFC DA_EC_ upon COMT, this mechanism would be predicted to impact the duration of the DA response to MA more so in this region than within NAC. However, arguing against a major role for drug-induced deficits in COMT in mediating the sensitization of MA-induced DA release is evidence that neither pharmacological inhibition of COMT nor null COMT mutation significantly impact amphetamine-induced increases in DA_EC_ within striatum or frontal cortex (Törnwall and Männistö, 1993; Törnwall et al., 1993; Tuomainen et al., 1996; Gogos et al., 1998; Männistö and Kaakkola, 1999; Huotari et al., 2002a,b).

Irrespective of the mechanisms at play, the fact that subchronic dosing with subtoxic MA elicited sensitization within both the NAC and mPFC is a finding in line with earlier reports for MA-experienced rodents (e.g., Stephans and Yamamoto, 1995; Zhang et al., 2001; Broom and Yamamoto, 2005; Lominac et al., 2012; Laćan et al., 2013; Le Cozannet et al., 2013; but see Ago et al., 2006, 2007, 2012). Moreover, the DA sensitization was time-independent, manifesting at 1 day post-treatment and persisting, unchanged, for at least 21 days (Figure 1). This finding distinguishes MA-induced DA sensitization from that produced by repeated cocaine or amphetamine, the latter two of which tends to grow with the passage of time during withdrawal (e.g., Paulson and Robinson, 1995; Vanderschuren and Kalivas, 2000). Nevertheless, the present results for MA-injected B6 mice are qualitatively similar to results from relatively recent studies, in which rats with a history of behavior-contingent vs. non-contingent intravenous MA exposure displayed MA-induced DA sensitization that manifested early in withdrawal (Lominac et al., 2012; Laćan et al., 2013; Le Cozannet et al., 2013). While requiring further study, particularly with respect to MA pharmacokinetics (see Segal and Kuczenski, 2006 for Discussion), the capacity of repeated MA to elicit time-independent DA sensitization within the NAC (and perhaps also within the mPFC) is qualitatively similar across rodent species, routes of delivery and contingency of delivery, which renders non-contingent models of MA administration well-suited for the study of the psychobiological consequences of subchronic MA exposure of relevance to MA abuse and the development of addiction (see also Laćan et al., 2013).

Peculiarly, the expression of MA-sensitized DA release within neither the NAC nor the mPFC of male B6 mice was not obviously related to drug effects upon basal DA_EC_ (Table 4). First, changes in DA_EC_ were only apparent in the long-term (Figure 2), while DA sensitization was manifest in early withdrawal. Second, MA history oppositely affected DA_EC_ in the mPFC (increase) and NAC (decrease), despite eliciting DA sensitization within both regions (Table 4). DA transmission within the NAC is highly implicated in mediating the incentive motivational properties of drugs and conditioned stimuli, as well as those for natural reinforcers, (e.g., Berridge and Robinson, 1998; Di Chiara, 1999; Robinson and Berridge, 2008; Wise, 2008; Blum et al., 2012). Indeed, the data from our neuropharmacological study of the NAC supports a bi-directional role for NAC DA in regulating the motivational valence of MA-conditioned environments, with elevated DA promoting the expression of conditioned approach and reduced DA eliciting conditioned avoidance (Figure 5A). In contrast, neuropharmacological manipulations of mPFC DA did not impact the magnitude or direction of conditioned behavior in our place-conditioning paradigm (Figure 5B). Such findings indicate that mPFC DA_EC_, particularly that within the prelimbic cortex (see histology in Figure 5B), does not actively regulate the recall of a drug-context associations or the conditioned incentive motivational properties of a drug-paired environment. This contrasts with the role for mPFC DA role in the acquisition of place-conditioning reported previously (e.g., Wilkinson et al., 1998; Hayen et al., 2014). The divergent effects of subchronic dosing with MA upon basal DA_EC_ within the NAC and mPFC indicate that distinct cellular or molecular mechanism(s) underpin the changes in basal vs. stimulated DA release in suchronic MA-treated animals within corticolimbic DA terminals. Whatever these mechanism(s) are that operate within the NAC and mPFC to impact DA_EC_ in the drugged vs. undrugged state (see below), their dysregulation by a suchronic history of MA injections is regionally selective and temporally distinct.

**Table 4:** **Comparison of the dopamine effects of subchronic, subtoxic methamphetamine (MA) vs. saline (SAL) treatment and the dopamine phenotype of MA-naïve MAHDR vs. MALDR mice**.

	**NAC**		**mPFC**	
	**MA- vs. SAL- treated**	**MAHDR vs. MALDR**	**MA- vs. SAL-treated**	**MAHDR vs. MALDR**
Basal extracellular content	↓ (protracted WD)	↓	↑ (protracted WD)	↓
MA-elicited dopamine release	↑	—	↑	↑
Total DAT protein	↓ (core)	↑ (shell and core)	—	n.d.
DAT function	—	n.d.	—	n.d.
Total D2 receptor protein	↓ (core)	↓ (shell)	—	—
D2/3 receptor function	↓	n.d.	↓ (protracted WD)	n.d.

*WD, withdrawal. ↑ denotes relative increase; ↓ denotes relative decrease;—denotes no change; n.d., denotes not determined*.

### NAC DA, MA preference and genetic vulnerability to MA intake

High-dose MA injection regimens are well-characterized to induce neurotoxicity within DA neurons in dorsal striatum, while sparing DA neurons within the NAC (c.f., Carvalho et al., 2012). However, evidence from studies using more moderate MA treatment regimens, including that herein (Figure 2B), indicate that subchronic, subtoxic MA can also lower NAC basal DA_EC_. The reduction in NAC basal DA_EC_ observed in male B6 mice herein is akin to that reported previously in male rats subjected to an identical MA injection regimen as that employed in the present study (Broom and Yamamoto, 2005). However, in contrast to this earlier study of rat, reduced basal DA_EC_ was apparent in our male mice only in protracted withdrawal and was paralleled by reduced DAT expression, but no discernable change in DAT function (as assessed by either Ed or the DA response to GBR-12909 infusion) (Table 4). Reduced striatal DAT binding is observed consistently in imaging studies of human MA addicts and MA-experienced non-human primates, even during protracted withdrawal (e.g., McCann et al., 1998; Sekine et al., 2001, 2003; Volkow et al., 2001a,b; Johanson et al., 2006; Groman et al., 2012). However, in MA-injected rats, reduced NAC basal DA_EC_ was reported to co-occur with *increased* DAT function and expression (Broom and Yamamoto, 2005). As this prior rat study did not examine for long-term changes in NAC DA levels or DAT expression/function, it is not known if MA-induced changes in DAT expression within the rat is biphasic with respect to time in withdrawal or if species differences exist for the long-term effects of repeated MA exposure upon NAC DA. Moreover, we and others have failed to detect pronounced changes in indices of NAC basal DA in rats with histories of intravenous MA (Schwendt et al., 2009; Lominac et al., 2012; Laćan et al., 2013; Le Cozannet et al., 2013). Thus, the role played by route of administration in the manifestation of MA-induced anomalies in DA_EC_ and DAT requires more systematic preclinical investigation.

In contrast to a recent study in non-human primates which failed to detect changes in D2R levels within the more ventral aspects of the striatum in MA-experienced subjects (Groman et al., 2012), our MA treatment regimen administered to male B6 mice reduced NAC D2R expression in long-term withdrawal (Figure 4). It is interesting to note that while this change in protein expression was late to manifest, an impairment in NAC D2/3R function was apparent very early in withdrawal prior to detectable changes in protein levels (Figure 4). Such data indicate a cause-effect relation between subchronic MA exposure and functional anomalies in D2/3Rs that are not necessarily related to gross alterations in total D2R protein expression, but could reflect changes in D3Rs, the latter of which we could not reliably detect with our immunoblotting procedures. Alternatively, as we measured total protein expression, the possibility existed that drug-elicited changes in the surface expression of proteins may have been masked by the study of whole tissue homogenate. D2/3Rs operate presynaptically as autoreceptors to inhibit both basal and impulse-dependent DA release (c.f. Ford, 2014). As such, the temporal relation between lowered D2/3R function and reduced DA_EC_ is not obvious at the present time. Nevertheless, these data extend the results of non-human primate studies (e.g., Groman et al., 2012) by demonstrating a cause-effect relation between subchronic MA history and NAC D2R expression/function that is not afforded in imaging studies of humans. This demonstration is of high clinical relevance given the purported link between polymorphisms in the gene encoding D2R, D2R hypofunctioning and MA addiction vulnerability, as well as addiction severity, in human and non-human primates (c.f., Blum et al., 2012; e.g., Lee et al., 2009; Groman et al., 2012).

Related to this latter point, female MAHDR mice exhibited lower NAC D2R expression, compared to their MALDR counterparts and the reduced NAC D2R expression was accompanied by lower basal DA_EC_, but higher DAT levels within this region (Table 4). The former two observations are consistent with the effects of subchronic MA upon the NAC of male B6 mice observed herein (Table 4) and are more, rather than less, consistent with recent work in non-human primates correlating low basal D2R availability within striatum to subsequent MA-taking (Groman et al., 2012). The higher DAT expression observed in MAHDR vs. MALDR female mice is consistent with earlier results for MA-injected male rats, which was suggested to contribute to the low DA_EC_ observed in MA-experienced animals (Broom and Yamamoto, 2005). Thus, drug-naïve or acute MA-injected female mice with a genetic predisposition to consume high amounts of MA exhibit DA anomalies within the NAC that are similar to, but not identical with, those reported in MA-experienced male rodents (Table 4). This later finding is interesting as earlier research clearly indicates that the striatum of female mice are much less sensitive to the neurotoxic effects of high-dose MA injection regimens, compared to males (Wagner et al., 1993; Yu and Wagner, 1994). Moreover, both female rodents (Morissette and Di Paolo, 1993a,b; Rivest et al., 1995; Bhatt and Dluzen, 2005; Ji and Dluzen, 2008) and humans (Lavalaye et al., 2000; Mozley et al., 2001; Staley et al., 2001) are reported to exhibit higher DAT expression or function than males. As we were limited in the total number and the sex of MAH/LDR animals available to study, it remains to be determined whether or not (1) MAHDR and MALDR mice differ in terms of DAT or DA autoreceptor function within the NAC (or other regions for that matter) and (2) the divergent D2R, DAT and DA_EC_ observed in MAH/LDR females mice occur also in males. Moreover, we could not determine how differences in basal DA_EC_, DAT, and D2R contribute to the divergent behavioral phenotypes of MAH/LDR mice nor could we determine the extent to which line differences in indices of DA function interact with sex to influence behavior. Nevertheless, from the similarities in NAC DA exhibited by suchronic MA-treated male and genetically vulnerable female mice (see Table 4), our findings herein resonate with results of recent studies for non-human primates indicating that either drug-induced or idiopathic reductions in NAC D2R expression and basal DA_EC_ may be critical biochemical triggers and/or predictors of subsequent high MA preference and intake (see Groman and Jentsch, 2013 for more detailed discussion). Extrapolating to the human condition, reduced striatal D2R binding reported in MA-addicted individuals (e.g., Volkow et al., 2001a; Lee et al., 2009) could very well reflect a combination of a pre-existing and drug-elicited hypo-DAergic state and further preclinical research is required in order to determine how idiopathies in ventral striatal D2R or basal DA_EC_ predict individual variation in MA preference and intake of relevance to the development of an addicted state.

### PFC DA and vulnerability to MA addiction

A subchronic history of subtoxic MA exposure increased mPFC DA_EC_ in B6 mice (Figure 2E), a finding in line with reduced tissue DOPAC reported in rats with a history of intravenous MA self-administration under short-access, operant procedures (Schwendt et al., 2009). Also consistent with this prior work, the MA-induced rise in mPFC DA_EC_ observed herein was found to be unrelated to indices of DAT function as we failed to detect saline-MA differences in the Ed for mPFC DA (an index of basal DA release/reuptake; Sam and Justice, 1996), total DAT expression or the capacity of intra-mPFC GBR-12909 infusions to elevate DA_EC_ (Figures 3C,D). Such data argue that the rise in mPFC DA_EC_ that manifests during protracted withdrawal from subchronic MA is not likely mediated by drug effects upon DAT and may very well reflect drug-elicited changes in other monoamine transporters, most notably NET. While we attempted to measure NET within the mPFC of our MA-injected mice to begin to address this possibility, the experiment was fraught with technical difficulties related to signal reliability of the available anti-NET primary antibodies, which prevented any firm conclusions on the matter. Future studies should assay the functional involvement of NET via the local infusion of selective reuptake inhibitors to probe the role for MA-induced changes in transporter function in the regulation of DA_EC_ within mPFC.

Consistent with the earlier report for MA self-administering rats (Schwendt et al., 2009), as well as rats treated non-contingently with drug (Liu et al., 2009), our subchronic MA injection regimen did not alter mPFC D2R expression (Figure 4D), supporting the notion that drug-elicited changes in frontal cortical DAT and D2R binding reported in MA addicts (e.g., Volkow et al., 2001a,b; Sekine et al., 2003), likely require extensive MA treatment histories in order to manifest (see Laćan et al., 2013 for Discussion). However, it is notable that MA-treated animals exhibited a reduced capacity of intra-mPFC sulpiride to elevate DA_EC_ and this effect manifested only in protracted withdrawal (Figure 4C). Thus, the possibility exists that the elevated DA_EC_ observed in the mPFC of MA-injected mice may reflect, at least in part, a progressive impairment in autoreceptor function within mPFC. To the best of our knowledge, these data are the first to characterize the effects of MA experience and withdrawal upon mPFC D2R function *in vivo* and we observe a cause-effect relation. Thus, while anomalies in frontal cortex function in MA addiction have been associated with low D2R availability in caudate (e.g., Volkow et al., 2001a), the present data indicate that subchronic drug experience is sufficient to produce local changes in D2/3R function and DA_EC_ within mPFC (primarily prelimbic cortex) that are predicted to impact cognitive control over drug-taking and –seeking early in the addiction process. As discussed above for the NAC, D2R hypofunctioning is highly associated with addiction, as well as other disorders characterized by motivational anomalies (c.f., Blum et al., 2012; Jentsch and Pennington, 2014). Moreover, reducing D2R function in both drug-naïve humans and laboratory animals elicits inhibitory control deficits that are akin to those observed in the clinical condition (e.g., Lee et al., 2007, 2009; Herold, 2010; Groman et al., 2011, 2012). Thus, while our attempt to probe the functional relevance of MA-induced changes in mPFC DA_EC_ and D2Rs failed to support a critical role for the expression of MA-conditioned approach/avoidance during early withdrawal (Figure 5B). Preliminary work from our laboratory indicates that the expression of MA-induced CPP in mice is highly resistant to extinction, persisting for weeks, even in the face of daily extinction training (Cohen, Barrett and Szumlinski, unpublished data). This raises the possibility that the impairment in mPFC D2/3R function produced by subchronic MA experience might underpin a deficiency in learning to inhibit forward approach behavior toward stimuli previously associated with MA and this possibility will be a topic of future research in our laboratory.

While low D2R availability within striatal structures is highly implicated in MA addiction vulnerability (c.f., Blum et al., 2012; Groman and Jentsch, 2013), there is little data supporting baseline D2R availability in frontal cortex with predisposition to addiction. In the present study, we did not detect any obvious relation between total D2R expression within mPFC and genetic vulnerability to high MA intake/preference (Figure 8C) and we were unsuccessful in our attempts to reliably detect DAT expression in the selected lines. Nevertheless, we did observe line differences in both basal and MA-stimulated DA_EC_ within the mPFC, which may of relevance to their divergent phenotypes. Opposite to MA-experienced B6 mice, MAHDR mice exhibited lower mPFC basal DA_EC_; however, both MA-experienced and MAHDR animals exhibited a more pronounced rise in MA-induced DA release within mPFC than their respective controls (Table 4). As DA release within mPFC contributes to the acquisition of Pavlovian and instrumental associations (e.g., Wilkinson et al., 1998), line differences in the DA responsiveness of the mPFC to acute MA might account, at least in part, for their divergent phenotypes when assessed in MA-induced place-preference and operant self-administration paradigms (Wheeler et al., 2009; Shabani et al., 2011, 2012a,b). That MAHDR mice exhibit “normal” MA-induced DA release within the NAC and greater MA-induced DA release within mPFC, in the face of lower basal content, suggests that the high MA-drinking phenotype of these animals (Wheeler et al., 2009) may reflect an attempt to supersede an allostatic state. Indeed, drug-naïve MAHDR animals exhibit signs of anhedonia, in that they exhibit lower instrumental responding for a palatable sweet solution despite exhibiting greater responding for MA reinforcement, compared to MALDR animals (Shabani et al., 2012a). Thus, the low baseline DA_EC_ (c.f. Wise, 2008), low basal D2R expression within the NAC (Blum et al., 2012), and/or dysregulated DA-5HT interactions within both the NAC and mPFC (e.g., Shirayama and Chaki, 2006) may all contribute to this presumed allostatic state in MAHDR mice that is theorized to underpin their addiction vulnerable phenotype.

### Anomalies in forebrain 5HT are correlates with high genetic vulnerability to MA intake

While the majority of this study focused on DA, MAHDR mice were reported to exhibit higher NAC *Slc6a4* mRNA expression than MALDR mice (Wheeler et al., 2009) and thus, we investigated also for genotype differences in indices of 5HT neurotransmission within the NAC and mPFC. Extending earlier results (Wheeler et al., 2009), MAHDR mice exhibited higher SERT expression within the NAC, but lower SERT expression within the mPFC, relative to MALDR mice (Table 5). Interestingly, the MAHDR-MALDR differences in SERT expression were inversely related to genotype differences in 5HT1BR within these two regions (Table 5). Thus, as observed for corticolimbic DA, genotype differences in indices of 5HT neurotransmission depended upon the forebrain region investigated.

**Table 5:** **Comparison of the protein expression of serotonin-related proteins within the nucleus accumbens (NAC) and medial prefrontal cortex (mPFC) of the MAHDR and MALDR selected lines**.

	**NAC**	**mPFC**
Basal extracellular content	MAHDR > MALDR	MAHDR = MALDR
MA-elicited serotonin release	MAHDR = MALDR	MAHDR < MALDR
Total SERT protein	MAHDR > MALDR (shell)	MAHDR < MALDR
Total 5HT1B protein	MAHDR < MALDR (core)	MAHDR > MALDR (n.s.)

*< denotes expression is less than; > denotes expression is greater than; = denotes no difference. n.s. indicates a strong trend for a genotypic difference that failed to reach statistical significance (i.e., p = 0.05–0.09)*.

In the NAC, higher basal 5HT_EC_ (Figure 6C) was coincident with higher SERT levels (Figures 8A,B) in MAHDR mice. Although we did not assay SERT function directly due to limited animal availability, we failed to detect line differences in the Ed for NAC 5HT using quantitative microdialysis procedures (Table 2), indicating no difference in 5HT uptake within this region (Sam and Justice, 1996). Thus, while the possibility may still exist that SERT is functioning sub-optimally within the NAC of MAHDR mice, a more parsimonious explanation is that the rise in SERT expression is merely a compensatory response to elevated 5HT_EC_, the latter of which results from 5HT1BR hypo-functioning (Table 5). Indeed, MA-stimulated monoamine release is primarily impulse-independent (e.g., Fleckenstein et al., 2007; Chen et al., 2009). Therefore, a perturbation in terminal 5HT1B autoreceptor function in MAHDR animals could underpin their elevated basal 5HT_EC_ levels, without necessarily influencing the capacity of MA to raise 5HT_EC_. As acute treatment with 2 mg/kg MA failed to elevate NAC 5HT_EC_ levels in either genotype (Figure 7C), it is difficult to discern a relation between the observed changes in 5HT_EC_, SERT and/or 5HT1BR expression to MA-induced 5HT release within the NAC. Nevertheless, the present data for MAH/LDR mice indicate that higher basal 5HT_EC_ and SERT, as well as lower 5HT1BR, expression within the NAC are correlates of high genetic vulnerability to MA intake, preference and reinforcement (see Wheeler et al., 2009; Shabani et al., 2011, 2012a,b) that are worthy of further exploration.

As was observed for forebrain DA, there were marked regional differences in the 5HT correlates of high vs. low genetic vulnerability to self-administer MA (Table 5). While MAHDR-MALDR differences were noted for NAC basal 5HT_EC_ content, no line differences were noted for mPFC basal 5HT_EC_ content or Ed. However, as observed for mPFC DA_EC_ (Table 4) marked genotype differences were apparent regarding MA-stimulated 5HT release in this region; however, in the latter case, MALDR mice were considerably more sensitive to MA than MAHDR animals (Figure 7D). In fact, the genotype difference in MA-induced 5HT release within mPFC was polar opposite that observed for DA release in this region (Figures 7B vs. 7D). This suggests a reciprocal interplay between these two monoamines systems, the basis of which cannot be discerned from the results of the present study. Nevertheless, the line differences in MA-induced 5HT release within the mPFC was associated with significant genotype differences in SERT and more moderate differences in 5HT1BR expression (Table 5). Notably, MAHDR mice displayed lower SERT and higher terminal autoreceptor expression, relative to MAHDR animals. Thus, the failure of MA to elevate 5HT_EC_ within the mPFC of MAHDR mice might relate to their lower levels of SERT, although the possibility that higher 5HT1BR autoreceptor tone might influence the amount of 5HT release cannot be negated at this time. Together, the above data for 5HT in MAH/LDR mice implicate anomalies in both basal and MA-induced changes in corticolimbic 5HT transmission in the propensity to develop a MA-addicted phenotype and research into individual variation in indices of mesocorticolimbic 5HT transmission and MA addiction, as well as a more systematic characterization of the effects of MA history upon forebrain 5HT, are warranted at both the clinical and preclinical levels in order to better understand the inter-relation between MA addiction vulnerability, addiction severity and 5HT.

## Conclusions

While a number of questions still remain, the results of the present study indicate that a history of subchronic, subtoxic MA is sufficient to produce a persistent dysregulation of indices of DA neurotransmission within the NAC and mPFC of mice. Moreover, we demonstrated that DA within the NAC, but not mPFC, actively regulates the expression of MA preference and mice with genetic vulnerability for high MA intake exhibit DA anomalies within the NAC, many of which are akin to those produced by subchronic MA experience. As the MA injection regimen employed herein attempted to model early drug experience, these data suggest an important role for idiopathic or drug-elicited anomalies in NAC DA for MA preference/intake. Moreover, as mice with a genetic vulnerability to high MA intake exhibit also anomalies in 5HT, particularly within the mPFC, implicates also mPFC 5HT neurotransmission in the etiology of MA addiction.

### Conflict of interest statement

The authors declare that the research was conducted in the absence of any commercial or financial relationships that could be construed as a potential conflict of interest.

## References

[B1] Ago Y. Nakamura S. Kajita N. Uda M. Hashimoto H. Baba A. (2007). Ritanserin reverses repeated methamphetamine-induced behavioral and neurochemical sensitization in mice. Synapse 61, 757–763 10.1002/syn.20421 17568413

[B2] Ago Y. Nakamura S. Uda M. Kajii Y. Abe M. Baba A. (2006). Attenuation by the 5-HT1A receptor agonist osemozotan of the behavioral effects of single and repeated methamphetamine in mice. Neuropharmacology 51, 914–922 10.1016/j.neuropharm.2006.06.001 16863654

[B3] Ago Y. Tanaka T. Kita Y. Tokumoto H. Takuma K. Matsuda T. (2012). Lithium attenuates methamphetamine-induced hyperlocomotion and behavioral sensitization via modulation of prefrontal monoamine release. Neuropharmacology 62, 1634–1639 10.1016/j.neuropharm.2011.10.004 22001792

[B4] Ares-Santos S. Granado N. Moratalla R. (2013). The role of dopamine receptors in the neurotoxicity of methamphetamine. J. Intern. Med. 273, 437–453 10.1111/joim.12049 23600399

[B5] Ary A. W. Lominac K. D. Wroten M. G. Williams A. R. Campbell R. R. Ben-Shahar O. (2013). Imbalances in prefrontal cortex *CC-*Homer1 versus -Homer2 expression promote cocaine-seeking behavior. J. Neurosci. 33, 8101–8113 10.1523/JNEUROSCI.1727-12.2013 23658151 PMC3704219

[B6] Berridge K. C. Robinson T. E. (1998). What is the role of dopamine in reward: hedonic impact, reward learning, or incentive salience? Brain Res. Rev. 28, 309–369 10.1016/S0165-0173(98)00019-8 9858756

[B7] Bhatt S. D. Dluzen D. E. (2005). Dopamine transporter function differences between male and female CD-1 mice. Brain Res. 1035, 188–195 10.1016/j.brainres.2004.12.013 15722058

[B8] Blum K. Chen A. L. Giordano J. Borsten J. Chen T. J. Hauser M. (2012). The addictive brain: all roads lead to dopamine. J. Psychoactive Drugs 44, 134–143 10.1080/02791072.2012.685407 22880541

[B9] Broom S. L. Yamamoto B. K. (2005). Effects of subchronic methamphetamine exposure on basal dopamine and stress-induced dopamine release in the nucleus accumbens shell of rats. Psychopharmacology 181, 467–476 10.1007/s00213-005-0007-6 15986185

[B10] Carvalho M. Carmo H. Costa V. M. Capela J. P. Pontes H. Remião F. (2012). Toxicity of amphetamines: an update. Arch. Toxicol. 86, 1167–1231 10.1007/s00204-012-0815-5 22392347

[B11] Chen J. C. Chen P. C. Chiang Y. C. (2009). Molecular mechanisms of psychostimulant addiction. Chang Gung Med. J. 32, 148–154 19403004

[B12] Cruickshank C. C. Dyer K. R. (2009). A review of the clinical pharmacology of methamphetamine. Addiction 104, 1085–1099 10.1111/j.1360-0443.2009.02564.x 19426289

[B13] Dean A. C. Groman S. M. Morales A. M. London E. D. (2013). An evaluation of the evidence that methamphetamine abuse causes cognitive decline in humans. Neuropsychopharmacology 38, 259–274 10.1038/npp.2012.179 22948978 PMC3527116

[B14] Di Chiara G. (1999). Drug addiction as dopamine-dependent associative learning disorder. Eur. J. Pharmacol. 375, 13–30 10.1016/S0014-2999(99)00372-6 10443561

[B15] Engleman E. A. Ingraham C. M. O'Brien C. E. McBride W. J. Murphy J. M. (2004). Effect of housing conditions on sulpiride-induced increases in extracellular dopamine levels in the nucleus accumbens of alcohol-preferring (P) rats. Brain Res. 1022, 247–250 10.1016/j.brainres.2004.06.069 15353236

[B16] Espana R. A. Jones S. R. (2013). Presynaptic dopamine modulation by stimulant self-administration. Front. Biosci. (Schol. Ed). 5, 261–276 23277050 10.2741/s371 PMC3733372

[B17] Fergusson D. M. Horwood L. J. Lynskey M. T. Madden P. A. (2003). Early reactions to cannabis predict later dependence. Arch. Gen. Psychiatry 60, 1033–1039 10.1001/archpsyc.60.10.1033 14557149

[B18] Fleckenstein A. E. Volz T. J. Riddle E. L. Gibb J. W. Hanson G. R. (2007). New insights into the mechanism of action of amphetamines. Annu. Rev. Pharmacol. Toxicol. 47, 681–698 10.1146/annurev.pharmtox.47.120505.105140 17209801

[B18a] Ford C. P. (2014). The role of D2-autoreceptors in regulating dopamine neuron activity and transmission. Neuroscience. [Epub ahead of print]. 10.1016/j.neuroscience.2014.01.025 24463000 PMC4108583

[B19] Fukakusa A. Nagai T. Mizoguchi H. Otsuka N. Kimura H. Kamei H. (2008). Role of tissue plasminogen activator in the sensitization of methamphetamine-induced dopamine release in the nucleus accumbens. J. Neurochem. 105, 436–444 10.1111/j.1471-4159.2007.05142.x 18036193

[B20] Galloway M. P. Wolf M. E. Roth R. H. (1986). Regulation of dopamine synthesis in the medial prefrontal cortex is mediated by release modulating autoreceptors: studies *in vivo*. J. Pharmacol. Exp. Ther. 236, 689–698 3081705

[B20a] Gogos J. A. Morgan M. Luine V. Santha M. Ogawa S. Pfaff D. (1998). Catechol-O-methyltransferase-deficient mice exhibit sexually dimorphic changes in catecholamine levels and behavior. Proc. Natl. Acad. Sci. U.S.A. 95, 9991–9996 10.1073/pnas.95.17.9991 9707588 PMC21449

[B21] Groman S. M. Jentsch J. D. (2013). Identifying the molecular basis of inhibitory control deficits in addictions: neuroimaging in non-human primates. Curr. Opin. Neurobiol. 23, 625–631 10.1016/j.conb.2013.03.001 23528268 PMC3731407

[B22] Groman S. M. Lee B. London E. D. Mandelkern M. A. James A. S. Feiler K. (2011). Dorsal striatal D2-like receptor availability covaries with sensitivity to positive reinforcement during discrimination learning. J. Neurosci. 31, 7291–7299 10.1523/JNEUROSCI.0363-11.2011 21593313 PMC3114883

[B21a] Groman S. M. Lee B. Seu E. James A. S. Feiler K. Mandelkern M. A. (2012). Dysregulation of D_2_-mediated dopamine transmission in monkeys after chronic escalating methamphetamine exposure. J. Neurosci. 32, 5843–5852 10.1523/JNEUROSCI.0029-12.2012 22539846 PMC3353813

[B23] Halpin L. E. Collins S. A. Yamamoto B. K. (2014). Neurotoxicity of methamphetamine and 3, 4-methylenedioxymethamphetamine. Life Sci. 97, 37–44 10.1016/j.lfs.2013.07.014 23892199 PMC3870191

[B24] Hayen A. Meese-Tamuri S. Gates A. Ito R. (2014). Opposing roles of prelimbic and infralimbic dopamine in conditioned cue and place preference. Psychopharmacology. [Epub ahead of print]. 10.1007/s00213-013-3414-0 24429871 PMC4039995

[B25] Herold C. (2010). NMDA and D2-like receptors modulate cognitive flexibility in a color discrimination reversal task in pigeons. Behav. Neurosci. 124, 381–390 10.1037/a0019504 20528082

[B26] Huotari M. Gogos J. A. Karayiorgou M. Koponen O. Forsberg M. Raasmaja A. (2002a). Brain catecholamine metabolism in catechol-O-methyltransferase (COMT)-deficient mice. Eur. J. Neurosci. 15, 246–256 10.1046/j.0953-816x.2001.01856.x 11849292

[B27] Huotari M. Santha M. Lucas L. R. Karayiorgou M. Gogos J. A. Männistö P. T. (2002b). Effect of dopamine uptake inhibition on brain catecholamine levels and locomotion in catechol-O-methyltransferase (COMT) disrupted mice. J. Pharmacol. Exp. Ther. 303, 1309–1316 10.1124/jpet.102.043042 12438556

[B28] Ikeda M. Ozaki N. Suzuki T. Kitajima T. Yamanouchi Y. Kinoshita Y. (2007). Possible association of beta-arrestin 2 gene with methamphetamine use disorder, but not schizophrenia. Genes Brain Behav. 6, 107–112 10.1111/j.1601-183X.2006.00237.x 17233643

[B29] Jentsch J. D. Pennington Z. T. (2014). Reward, interrupted: inhibitory control and its relevance to addictions. Neuropharmacology 76 (Pt B), 479–486 10.1016/j.neuropharm.2013.05.022 23748054 PMC4023480

[B30] Ji J. Dluzen D. E. (2008). Sex differences in striatal dopaminergic function within heterozygous mutant dopamine transporter knock-out mice. J. Neural Transm. 115, 809–817 10.1007/s00702-007-0017-0 18197357

[B31] Johanson C. E. Frey K. A. Lundahl L. H. Keenan P. Lockhart N. Roll J. (2006). Cognitive function and nigrostriatal markers in abstinent methamphetamine abusers. Psychopharmacology 185, 327–338 10.1007/s00213-006-0330-6 16518646

[B32] Karoum F. Chrapusta S. J. Egan M. F. (1994). 3-Methoxytyramine is the major metabolite of released dopamine in the rat frontal cortex: reassessment of the effects of antipsychotics on the dynamics of dopamine release and metabolism in the frontal cortex, nucleus accumbens, and striatum by a simple two pool model. J. Neurochem. 63, 972–979 10.1046/j.1471-4159.1994.63030972.x 7914228

[B33] Kuhn D. M. Angoa-Pérez M. Thomas D. M. (2011). Nucleus accumbens invulnerability to methamphetamine neurotoxicity. ILAR J. 52, 352–365 10.1093/ilar.52.3.352 23382149 PMC4798000

[B34] Laćan G. Hadamitzky M. Kuczenski R. Melega W. P. (2013). Alterations in the striatal dopamine system during intravenous methamphetamine exposure: effects of contingent and noncontingent administration. Synapse 67, 476–488 10.1002/syn.21654 23417852 PMC4019070

[B35] Lavalaye J. Booij J. Reneman L. Harbraken J. B. van Royen E. A. (2000). Effect of age and gender on dopamine transporter imaging with [123I] FP-CIT SPET in healthy volunteers. Eur. J. Nucl. Med. 27, 867–869 10.1007/s002590000279 10952500

[B36] Le Cozannet R. Markou A. Kuczenski R. (2013). Extended-access, but not limited-access, methamphetamine self-administration induces behavioral and nucleus accumbens dopamine response changes in rats. Eur. J. Neurosci. 38, 3487–3495 10.1111/ejn.12361 24112125 PMC3841010

[B37] Lee B. Groman S. London E. D. Jentsch J. D. (2007). Dopamine D2/D3 receptors play a specific role in the reversal of a learned visual discrimination in monkeys. Neuropsychopharmacology 32, 2125–2134 10.1038/sj.npp.1301337 17299511

[B38] Lee B. London E. D. Poldrack R. A. Farahi J. Nacca A. Monterosso J. R. (2009). Striatal dopamine d2/d3 receptor availability is reduced in methamphetamine dependence and is linked to impulsivity. J. Neurosci. 29, 14734–11440 10.1523/JNEUROSCI.3765-09.2009 19940168 PMC2822639

[B39a] Liu K. Steketee J. D. (2011). Repeated exposure to cocaine alters medial prefrontal cortex dopamine D_2_-like receptor modulation of glutamate and dopamine neurotransmission within the mesocorticolimbic system. J. Neurochem. 119, 332–341 10.1111/j.1471-4159.2011.07362.x 21692802

[B39] Liu X. Chang L. Vigorito M. Kass M. Li H. Chang S. L. (2009). Methamphetamine-induced behavioral sensitization is enhanced in the HIV-1 transgenic rat. J. Neuroimmune Pharmacol. 4, 309–316 10.1007/s11481-009-9160-8 19444617

[B40] Lominac K. D. Sacramento A. D. Szumlinski K. K. Kippin T. E. (2012). Distinct neurochemical adaptations within the nucleus accumbens produced by a history of self-administered versus non-contingently administered intravenous methamphetamine. Neuropsychopharmacology 37, 707–722 10.1038/npp.2011.248 22030712 PMC3260984

[B40a] Männistö P. T. Kaakkola S. (1999). Catechol-O-methyltransferase (COMT): biochemistry, molecular biology, pharmacology, and clinical efficacy of the new selective COMT inhibitors. Pharmacol. Rev. 51, 593–628 10581325

[B40b] Matsumoto M. Weickert C. S. Akil M. Lipska B. K. Hyde T. M. Herman M. M. (2003). Catechol O-methyltransferase mRNA expression in human and rat brain: evidence for a role in cortical neuronal function. Neuroscience 116, 127–137 10.1016/S0306-4522(02)00556-0 12535946

[B41] McCann U. D. Ricaurte G. A. (2004). Amphetamine neurotoxicity: accomplishments and remaining challenges. Neurosci. Biobehav. Rev. 27, 821–826 10.1016/j.neubiorev.2003.11.003 15019431

[B42] McCann U. D. Wong D. F. Yokoi F. Villemagne V. Dannals R. F. Ricaurte G. A. (1998). Reduced striatal dopamine transporter density in abstinent methamphetamine and methcathinone users: evidence from positron emission tomography studies with [11C]WIN-35,428. J. Neurosci. 18, 8417–8422 9763484 10.1523/JNEUROSCI.18-20-08417.1998 PMC6792853

[B43] Morissette M. Di Paolo T. (1993a). Effect of chronic estradiol and progesterone treatments of ovariectomized rats on brain dopamine uptake sites. J. Neurochem. 60, 1876–1883 10.1111/j.1471-4159.1993.tb13415.x 8473903

[B44] Morissette M. Di Paolo T. (1993b). Sex and estrous cycle variations of rat striatal dopamine uptake sites. Neuroendocrinology 58, 16–22 10.1159/000126507 8264850

[B45] Morita Y. Ujike H. Tanaka Y. Kishimoto M. Okahisa Y. Kotaka T. (2008). The glycine transporter 1 gene (GLYT1) is associated with methamphetamine-use disorder. Am. J. Med. Genet. B Neuropsychiatr. Genet. 147B, 54–58 10.1002/ajmg.b.30565 17582620

[B46] Mozley L. H. Gur R. C. Mozley P. D. Gur R. E. (2001). Striatal dopamine transporters and cognitive functioning in healthy men and women. Am. J. Psychiatry 158, 1492–1499 10.1176/appi.ajp.158.9.1492 11532737

[B47] Paulson P. E. Robinson T. E. (1995). Amphetamine-induced time-dependent sensitization of dopamine neurotransmission in the dorsal and ventral striatum: a microdialysis study in behaving rats. Synapse 19, 56–65 10.1002/syn.890190108 7709344 PMC1859849

[B48] Paxinos G. Franklin K. B. J. (2007). The Mouse Brain in Stereotaxic Coordinates. Maryland Heights, MO: Academic Press

[B49] Petrakis I. L. Limoncelli D. Gueorguieva R. Jatlow P. Boutros N. N. Trevisan L. (2004). Altered NMDA glutamate receptor antagonist response in individuals with a family vulnerability to alcoholism. Am. J. Psychiatry 161, 1776–1782 10.1176/appi.ajp.161.10.1776 15465973

[B50] Phillips T. J. Kamens H. M. Wheeler J. M. (2008). Behavioral genetic contributions to the study of addiction-related amphetamine effects. Neurosci. Biobehav. Rev. 32, 707–759 10.1016/j.neubiorev.2007.10.008 18207241 PMC2360482

[B51] Pierce R. C. Kalivas P. W. (1997). Repeated cocaine modifies the mechanism by which amphetamine releases dopamine. J. Neurosci. 17, 3254–3261 9096158 10.1523/JNEUROSCI.17-09-03254.1997 PMC6573637

[B52] Popova N. K. Gilinskii M. A. Amstislavskaya T. G. (2004). Effect of monoamine oxidase gene knockout on dopamine metabolism in mouse brain structures. Bull. Exp. Biol. Med. 137, 382–384 10.1023/B:BEBM.0000035137.97552.ab 15452609

[B54] Rivest R. Falardeau P. Di Paolo T. (1995). Brain dopamine transporter: gender differences and effects of chronic haloperidol. Brain Res. 692, 269–272 10.1016/0006-8993(95)00611-S 8548314

[B54a] Robinson T. E. Becker J. B. (1986). Enduring changes in brain and behavior produced by chronic amphetamine administration: a review and evaluation of animal models of amphetamine psychosis. Brain Res. 396, 157–198 10.1016/0165-0173(86)90002-0 3527341

[B55] Robinson T. E. Berridge K. C. (2008). The incentive sensitization theory of addiction: some current issues. Philos. Trans. R. Soc. Lond. B Biol. Sci. 363, 3137–3146 10.1098/rstb.2008.0093 18640920 PMC2607325

[B56] Rusyniak D. E. (2011). Neurologic manifestations of chronic methamphetamine abuse. Neurol. Clin. 29, 641–655 10.1016/j.ncl.2011.05.004 21803215 PMC3148451

[B57] Sam P. M. Justice J. B. Jr. (1996). Effect of general microdialysis-induced depletion on extracellular dopamine. Anal. Chem. 68, 724–728 10.1021/ac950754+ 8779439

[B58] Santiago M. Machado A. Cano J. (1993). Regulation of prefrontal cortical dopamine release by dopamine receptor agonists and antagonists. Eur. J. Pharmacol. 239, 83–91 10.1016/0014-2999(93)90979-R 7901031

[B59] Schuckit M. A. Tipp J. E. Smith T. L. Wiesbeck G. A. Kalmijn J. (1997). The relationship between Self-Rating of the Effects of alcohol and alcohol challenge results in ninety-eight young men. J. Stud. Alcohol 58, 397–404 9203121 10.15288/jsa.1997.58.397

[B60] Schwendt M. Rocha A. See R. E. Pacchioni A. M. McGinty J. F. Kalivas P. (2009). Extended methamphetamine self-administration in rats results in a selective reduction of dopamine transporter levels in the prefrontal cortex and dorsal striatum not accompanied by marked monoaminergic depletion. J. Pharmacol. Exp. Ther. 331, 555–562 10.1124/jpet.109.155770 19648469 PMC2775260

[B61] Segal D. S. Kuczenski R. (2006). Human methamphetamine pharmacokinetics simulated in the rat: single daily intravenous administration reveals elements of sensitization and tolerance. Neuropsychopharmacology 31, 941–955 10.1038/sj.npp.1300865 16123749

[B62] Sekine Y. Iyo M. Ouchi Y. Matsunaga T. Tsukada H. Okada H. (2001). Methamphetamine-related psychiatric symptoms and reduced brain dopamine transporters studied with PET. Am. J. Psychiatry 158, 1206–1214 10.1176/appi.ajp.158.8.1206 11481152

[B63] Sekine Y. Minabe Y. Ouchi Y. Takei N. Iyo M. Nakamura K. (2003). Association of dopamine transporter loss in the orbitofrontal and dorsolateral prefrontal cortices with methamphetamine-related psychiatric symptoms. Am. J. Psychiatry 160, 1699–1701 10.1176/appi.ajp.160.9.1699 12944350

[B64] Sesack S. R. Hawrylak V. A. Matus C. Guido M. A. Levey A. I. (1998). Dopamine axon varicosities in the prelimbic division of the rat prefrontal cortex exhibit sparse immunoreactivity for the dopamine transporter. J. Neurosci. 18, 2697–2708 9502827 10.1523/JNEUROSCI.18-07-02697.1998 PMC6793120

[B65] Shabani S. Dobbs L. K. Ford M. M. Mark G. P. Finn D. A. Phillips T. J. (2012a). A genetic animal model of differential sensitivity to methamphetamine reinforcement. Neuropharmacology 62, 2169–2177 10.1016/j.neuropharm.2012.01.002 22280875 PMC3320769

[B66] Shabani S. Mckinnon C. S. Cunningham C. L. Phillips T. J. (2012b). Profound reduction in sensitivity to the aversive effects of methamphetamine in mice bred for high methamphetamine intake. Neuropharmacology 62, 1134–1141 10.1016/j.neuropharm.2011.11.005 22118879 PMC3297479

[B67] Shabani S. McKinnon C. S. Reed C. R. Cunningham C. L. Phillips T. J. (2011). Sensitivity to rewarding or aversive effects of methamphetamine determines methamphetamine intake. Genes Brain Behav. 10, 625–636 10.1111/j.1601-183X.2011.00700.x 21554535 PMC3320762

[B67a] Shirayama Y. Chaki S. (2006). Neurochemistry of the nucleus accumbens and its relevance to depression and antidepressant action in rodents. Curr. Neuropharmacol. 4, 277–291 10.2174/157015906778520773 18654637 PMC2475798

[B68] Shoblock J. R. Maisonneuve I. M. Glick S. D. (2003). Differences between d-methamphetamine and d-amphetamine in rats: working memory, tolerance, and extinction. Psychopharmacology 170, 150–156 10.1007/s00213-003-1522-y 12774190

[B69] Staley J. K. Krishnan-Sarin S. Zoghbi S. Tamagnan G. Fujita M. Seibyl J. P. (2001). Sex differences in [123I]beta-CIT SPECT measures of dopamine and serotonin transporter availability in healthy smokers and nonsmokers. Synapse 41, 275–284 10.1002/syn.1084 11494398

[B70] Stephans S. E. Yamamoto B. K. (1995). Effect of repeated methamphetamine administration on dopamine and glutamate efflux in rat prefrontal cortex. Brain Res. 700, 99–106 10.1016/0006-8993(95)00938-M 8624733

[B71] Sulzer D. Sonders M. S. Poulsen N. W. Galli A. (2005). Mechanisms of neurotransmitter release by amphetamines: a review. Prog. Neurobiol. 75, 406–433 10.1016/j.pneurobio.2005.04.003 15955613

[B72] Szumlinski K. K. Liu A. Penzner J. H. Lominac K. D. (2007). Protracted “pro-addictive” phenotype produced by pre-adolescent phenylpropanolamine. Neuropsychopharmacology 32, 1760–1773 10.1038/sj.npp.1301306 17251912

[B72a] Törnwall M. Männistö P. T. (1993). Effects of three types of catechol O-methylation inhibitors on L-3,4-dihydroxyphenylalanine-induced circling behaviour in rats. Eur. J. Pharmacol. 250, 77–84 10.1016/0014-2999(93)90623-P 8119326

[B72b] Törnwall M. Tuomainen P. Männistö P. T. (1993). Modulation of rat brain endogenous dopamine metabolism by new inhibitors of catechol O-methyltransferase. Eur. J. Pharmacol. 239, 39–45 10.1016/0014-2999(93)90973-L 8223912

[B72c] Tuomainen P. Törnwall M. Männistö P. T. (1996). Minor effect of tolcapone, a catechol-O-methyltransferase inhibitor, on extracellular dopamine levels modified by amphetamine or pargyline: a microdialysis study in anaesthetized rats. Pharmacol. Toxicol. 78, 392–396 10.1111/j.1600-0773.1996.tb00224.x 8829199

[B73] Uhl G. R. Drgon T. Liu Q. R. Johnson C. Walther D. Komiyama T. (2008). Genome-wide association for methamphetamine dependence: convergent results from 2 samples. Arch. Gen. Psychiatry 65, 345–355 10.1001/archpsyc.65.3.345 18316681

[B73a] Ujike H. Onoue T. Akiyama K. Hamamura T. Otsuki S. (1989). Effects of selective D-1 and D-2 dopamine antagonists on development of methamphetamine-induced behavioral sensitization. Psychopharmacology (Berl.) 98, 89–92 10.1007/BF00442011 2498964

[B74] United Nations Office on Drugs and Crime. (2011). World Drug Report. New York, NY: United Nations.

[B75] Vanderschuren L. J. Kalivas P. W. (2000). Alterations in dopaminergic and glutamatergic transmission in the induction and expression of behavioral sensitization: a critical review of preclinical studies. Psychopharmacology 151, 99–120 10.1007/s002130000493 10972458

[B76] Volkow N. D. Chang L. Wang G. J. Fowler J. S. Ding Y. S. Sedler M. (2001b). Low level of brain dopamine D2 receptors in methamphetamine abusers: association with metabolism in the orbitofrontal cortex. Am. J. Psychiatry 158, 2015–2021 10.1176/appi.ajp.158.12.2015 11729018

[B77] Volkow N. D. Chang L. Wang G. J. Fowler J. S. Franceschi D. Sedler M. (2001a). Loss of dopamine transporters in methamphetamine abusers recovers with protracted abstinence. J. Neurosci. 21, 9414–9418 11717374 10.1523/JNEUROSCI.21-23-09414.2001 PMC6763886

[B77a] Wagner G. C. Tekirian T. L. Cheo C. T. (1993). Sexual differences in sensitivity to methamphetamine toxicity. J. Neural. Transm. Gen. Sect. 93, 67–70 10.1007/BF01244939 8373556

[B78] Westerink B. H. C. Cremers T. I. H. F. (eds.). (2007). Handbook of Microdialysis: Methods, Applications and Clinical Aspects. London: Academic Press

[B79] Wheeler J. M. Reed C. Burkhart-Kasch S. Li N. Cunningham C. L. Janowsky A. (2009). Genetically correlated effects of selective breeding for high and low methamphetamine consumption. Genes Brain Behav. 8, 758–771 10.1111/j.1601-183X.2009.00522.x 19689456 PMC2783502

[B80] Wilkinson L. S. Humby T. Killcross A. S. Torres E. M. Everitt B. J. Robbins T. W. (1998). Dissociations in dopamine release in medial prefrontal cortex and ventral striatum during the acquisition and extinction of classical aversive conditioning in the rat. Eur. J. Neurosci. 10, 1019–1026 10.1046/j.1460-9568.1998.00119.x 9753169

[B81] Wise R. A. (2008). Dopamine and reward: the anhedonia hypothesis 30 years on. Neurotox. Res. 14, 169–183 10.1007/BF03033808 19073424 PMC3155128

[B82] Yamamoto B. K. Bankson M. G. (2005). Amphetamine neurotoxicity: cause and consequence of oxidative stress. Crit. Rev. Neurobiol. 17, 87–117 10.1615/CritRevNeurobiol.v17.i2.30 16808729

[B83] Yang M. H. Jung M. S. Lee M. J. Yoo K. H. Yook Y. J. Park E. Y. (2008a). Gene expression profiling of the rewarding effect caused by methamphetamine in the mesolimbic dopamine system. Mol. Cells 26, 121–130 18594179

[B84] Yang M. H. Kim S. Jung M. S. Shim J. H. Ryu N. K. Yook Y. J. (2008b). Proteomic analysis of methamphetamine-induced reinforcement processes within the mesolimbic dopamine system. Addict. Biol. 13, 287–294 10.1111/j.1369-1600.2007.00090.x 18279499

[B84a] Yu Y. L. Wagner G. C. (1994). Influence of gonadal hormones on sexual differences in sensitivity to methamphetamine-induced neurotoxicity. J. Neural Transm. Park. Dis. Dement. Sect. 8, 215–221 10.1007/BF02260942 7538305

[B85] Zhang Y. Loonam T. M. Noailles P. A. Angulo J. A. (2001). Comparison of cocaine- and methamphetamine-evoked dopamine and glutamate overflow in somatodendritic and terminal field regions of the rat brain during acute, chronic, and early withdrawal conditions. Ann. N. Y. Acad. Sci. 937, 93–120 10.1111/j.1749-6632.2001.tb03560.x 11458542

